# Comparative analysis of genomic prediction approaches for multiple time-resolved traits in maize

**DOI:** 10.1007/s00122-026-05162-4

**Published:** 2026-02-06

**Authors:** David Hobby, Robin Lindner, Alain J. Mbebi, Hao Tong, Zoran Nikoloski

**Affiliations:** 1 Bioinformatics Department, Institute of Biochemistry and Biology, University of Potsdam, Karl-Liebknecht-Str. 24-25, 14476 Potsdam, Brandenburg Germany; 2 Systems Biology and Mathematical Modeling, Max Planck Institute of Molecular Plant Physiology, Am Mühlenberg 1, 14476 Potsdam, Brandenburg Germany; 3 Plant Phenomics Research Centre, Academy for Advanced Interdisciplinary Studies, Nanjing Agricultural University, Nanjing, 210095 Jiangsu Province China

## Abstract

Ability to accurately predict multiple growth-related traits over plant developmental trajectories has the potential to revolutionize crop breeding and precision agriculture. Despite increased availability of time-resolved data for multiple traits from high-throughput phenotyping platforms of model plants and crops, genomic prediction is largely applied independently to a small number of traits, often neglecting their dynamics. Here, we compared and contrasted the performance of MegaLMM and dynamicGP as well as hybrid variants, using MegaLMM in place of RR-BLUP for component matrix prediction, which can handle high-dimensional temporal data for multi-trait genomic prediction. The comparative analysis made use of time series for 50 geometric, color, and texture traits in a maize multi-parent advanced generation inter-cross (MAGIC) population. The performance of the approaches was assessed using snapshot and longitudinal accuracy, quantified as the Pearson correlation (PCC) and mean squared error (MSE), thereby providing insight into the ability to predict multiple traits at a single time point or the dynamics of individual traits over the considered time domain, respectively. We found that MegaLMM outperforms dynamicGP in terms of both snapshot and longitudinal PCC over an observed time interval, but not in terms of snapshot MSE. We also analyzed the characteristics of trait developmental trajectories associated with predictive performance. This study goes further to demonstrate that dynamicGP is the only time-dependent genomic prediction approach which can forecast multiple traits beyond the set of training time points and paves the way for careful investigation of factors that affect the capacity to predict dynamics of multiple traits from genetic markers alone.

**Supplementary Information:**

The online version contains supplementary material available at 10.1007/s00122-026-05162-4.

## Introduction

The plant phenome is a complex outcome shaped by the dynamic interplay of genetic and environmental factors over time. Classical genomic prediction (GP) models have predominantly focused on predicting traits measured at a single time point, providing a snapshot of one or multiple traits in a particular developmental stage for a population of genotypes. However, the advent of high-throughput phenotyping (HTP) technologies has enabled the collection of time-resolved phenotypic data representing hundreds of traits, providing an opportunity to: (i) study the trait dynamics over the course of development, (ii) better understand the relationships between these traits during the plant life cycle, and (iii) use these traits in predicting final agronomically relevant phenotypes, like yield.

Simultaneous prediction of multiple traits, characterizing a part of the entire phenome of a plant, can be achieved by multi-trait GP models (Mbebi et al. 2025; Melsen et al. 2025). These models make use of genetic and phenotypic correlations among the considered traits, often leading to more accurate predictions compared to traditional single-trait approaches (Bhatta et al. 2020; Jia and Jannink 2012; Arojju et al. 2020; Shahi et al. 2022). The improved accuracy of multi-trait GP models is largely due to improved predictions of low-heritability traits that are correlated to high-heritability traits, which can be better predicted from genomic data. However, classical multi-trait genomic prediction (GP) models are computationally demanding, usually addressed by selecting a subset of traits to limit the number of parameters in the model. In addition, with one exception (Baba et al. 2020), the classical multi-trait GP models have not yet tackled the simultaneous prediction of multiple traits over several time points. Access to and application of such models are of great relevance in practice since they can significantly reduce the efforts for phenotyping of large-scale populations. For clarity we refer to classical multi-trait GP models as multi-variate GP (MVGP), irrespective of whether these variables denote a single trait at multiple time points or multiple traits at a single or multiple time points; the use of models to predict multiple distinct phenotypic traits is referred to as multi-trait GP (MTGP).

Using the premise of the classical GP approach, there are four potential routes to predicting a phenome across time using genomic data (Hobby et al. 2025): (1) single-trait models at individual time points (ST-STP), concerned with predicting each trait at a separate time point. This approach fails to exploit information which may be available from other traits or time points. (2) multi-trait models at a single time point (MT-STP); here, a MVGP model is built for multiple traits jointly at each time point. Although this approach is an improvement over (1) in that it exploits the within-time-point covariance between traits, it neglects the valuable relationships among traits across different time points. Note that this is distinct from, but methodologically related to, the prediction of the same set of traits and genotypes across multiple years (Montesinos-López et al. 2021). (3) single trait across a given range of time points (ST-MTP); predicts one trait over multiple time points within a MVGP model. This approach, too, is an improvement over (1), by making use of inter-time-point covariance within a single trait, but neglects the inter-trait correlations which can contribute considerably to the final predictive ability (Mbebi et al. 2025). Finally, (4) MVGP models for multiple traits across a given range of time points (MT-MTP); this is the most comprehensive approach that involves a simultaneous analysis of all traits of interest across all investigated time points. With the ability to capture both inter-trait and inter-time-point correlations, we hypothesize that predictions from approach (4) have the greatest promise to showcase the advantage of genomic prediction to forecast multiple traits at once using genetic markers alone. However, using approach (4) also requires filling a knowledge gap in devising specialized computational and modeling tools that can handle high-dimensional time-resolved data in a genomic prediction setting. Moreover, it should be noted that while the absolute number of models trained in MT-STP, ST-MTP, and MT-MTP configurations is smaller compared to ST-STP, these models are larger, with more parameters to be determined, may require more advanced computational approaches, are more computationally demanding, and take longer to fit than single-trait models for each of the trait-time-point pairs.

An additional dimension of classification for longitudinal GP modeling is between direct vs. indirect prediction of phenotypes (Hobby et al. 2025). Direct longitudinal GP models correspond to classical single or MVGP methods (Mbebi et al. 2025) applied to longitudinal data in one of the previously mentioned configurations. An example of this is seen in Adak et al. (2023) where individual RR-BLUP models were used at each time point to predict RGB color traits. Indirect longitudinal GP models abstract away from the original phenotypic data either by (i) fitting parametric or nonparametric function(s) to the developmental trajectory of a given trait within a population of genotypes (Toda et al. 2024; Lyra et al. 2020); or (ii) applying a dimensionality reduction method such as principle component analysis to the data (Lyra et al. 2020; Rooney et al. 2022). Subsequently, classical GP methods are applied to the set of coefficients or principal components obtained. Further, the methods that model developmental trajectories are dynamic methods, while the direct methods and dimensionality reduction-based methods are static methods. The dynamic methods have the advantage of being able to forecast traits beyond the last available time point in the training set; attempting the same with static methods would simply rely on correlations against the final available time point.

There have been significant advances for single-trait GP models with data from individual time points, and they are widely applied in practice (Tong and Nikoloski 2021). While several classes of MVGP models have been proposed, facilitating their usage with traits at a single time point, the wider application of these models is precluded largely due to computational demands (Mbebi et al. 2025). Single-trait GP models cannot include additional phenotypic data from related traits as “secondary” traits and thus the “focal” trait is solely predicted on the basis of genomic data. In contrast, MVGP models can make use of several potential cross-validation (CV) configurations, including  the longitudinal CV nomenclature from Hobby et al. (2025). MVGP models can either rely solely on genomic data (CV1) or can incorporate: (i) the same trait(s), measured in the same or different environments, or (ii) different but correlated “secondary” traits (CV2) (Melsen et al. 2025), often leading to enhanced prediction accuracy.

A notable exception within existing MVGP models is the mega-scale linear mixed models (MegaLMM) (Runcie et al. 2021) which can elegantly handle complex, high-dimensional phenotypic data, such as those generated in HTP facilities. MegaLMM supports both CV1 and the inclusion of secondary traits to assist the prediction of focal traits in a CV2 prediction setup, and has proven superior to Genomic Best Linear Unbiased Prediction (GBLUP) in both single- and multi-environment trials when predicting traits like plant height, grain yield, days to silking, and anthesis-silking interval in maize (Runcie et al. 2021). Due to its latent factorization step, MegaLMM can handle negatively correlated traits in contrast to classical MVGP methods based on vectorization; therefore, MegaLMM foregoes the need of pre-selecting traits based on positive correlation. Further, MegaLMM models applied to data from single field trials outperformed both GBLUP and BayesB in predicting 18 maize stalk quality traits (Hu et al. 2022). These models utilized less labor-intensive morphological traits (*i.e.,* secondary traits) to predict stalk quality (*i.e.,* focal traits), which are more challenging to measure. Although MegaLMM has previously been applied to field data obtained with drone technology for HTP (Runcie et al. 2021), it has not yet been applied to controlled environment HTP, nor to time-series data.

Although there are several indirect longitudinal GP approaches which have tackled the problem of predicting trait dynamics across time, these approaches have been limited to one or two traits (Momen et al. 2019; Toda et al. 2021; Rooney et al. 2022), or have incorporated a set of secondary traits to predict a single focal trait (Baba et al. 2020; Sakurai et al. 2023). To the best of our knowledge, there is a single approach, termed dynamicGP, that has tackled the problem of simultaneously predicting the dynamics of multiple traits, resulting in multi-trait models across a given range of time points (Hobby et al. 2025). DynamicGP combines Dynamic Mode Decomposition (DMD) (Kutz et al. 2016) with GP, by using the (trait $\times$ time) matrix **X** as input in a DMD algorithm to derive a time-invariant operator (matrix) **A**. This time-invariant operator is subsequently used for the prediction of multiple traits at one time from their value at a preceding time point. This is possible since the operator, **A** captures the dynamics between traits at consecutive time points. DynamicGP relies on training models based on the widely used single-trait genomic prediction method Ridge-Regression Best Linear Unbiased Prediction (RR-BLUP) (Endelman 2011) to predict the intermediate components **R** and $\boldsymbol{\Phi }$ of DMD based on Schur decomposition (Schur-DMD) (Thitsa et al. 2021) with genomic data (Hobby et al. 2025). As a result, dynamicGP predicts the dynamics of traits over time, rather than at a single time point. Essentially, this approach predicts the evolution of traits by predicting the matrix **A** for genotypes that were not used in the training set. DynamicGP has two variants: (1) iterative, where the true trait values at each time point are used to predict the values at the time point directly following it, and, of more practical value (2) recursive, where the trait values at the initial (first) time point are used to predict the second, which are then used recursively to predict the traits at all subsequent time points. Within the above established classification framework, both MegaLMM and dynamicGP represent indirect MT-MTP methods since they apply dimension reduction methods—latent factorization in the case of MegaLMM, and DMD in the case of dynamicGP—to the phenotypic data to obtain lower-dimensional representations of the phenotypes. These latent data representations are then predicted using classical GP methods, and in turn used to make predictions of the original phenotypes. A major difference between MegaLMM and dynamicGP is that MegaLMM does not explicitly seek to capture the dynamics or model developmental trajectories, rendering it a indirect static method; in contrast, dynamicGP explicitly captures the dynamics, rendering it an indirect dynamic method which performs dimension reduction and models the dynamics of multiple traits simultaneously.

One possible limitation of dynamicGP, which uses RR-BLUP to predict the intermediate matrices of the Schur-DMD algorithm, is that a separate model is required for each element of the predicted matrices, therefore not explicitly modeling inter-element covariance. More specifically, given the number of included singular values and vectors *r*, predicting the dynamics of *n* traits requires building *r* $\times$ *n* + *r*$^{2}$ RR-BLUP models (see Materials and Methods) of the individual matrix elements. Here, rather than training an RR-BLUP model for each individual element of the DMD components, we aim to examine the extent to which a MegaLMM model can exploit inter-element covariance and improve the prediction accuracy of the building blocks of dynamicGP. In addition, we provide a systematic performance comparison of dynamicGP and several comparable configurations of MegaLMM to predict multiple plant traits measured in a controlled environment of an HTP facility.

**Fig. 1:**
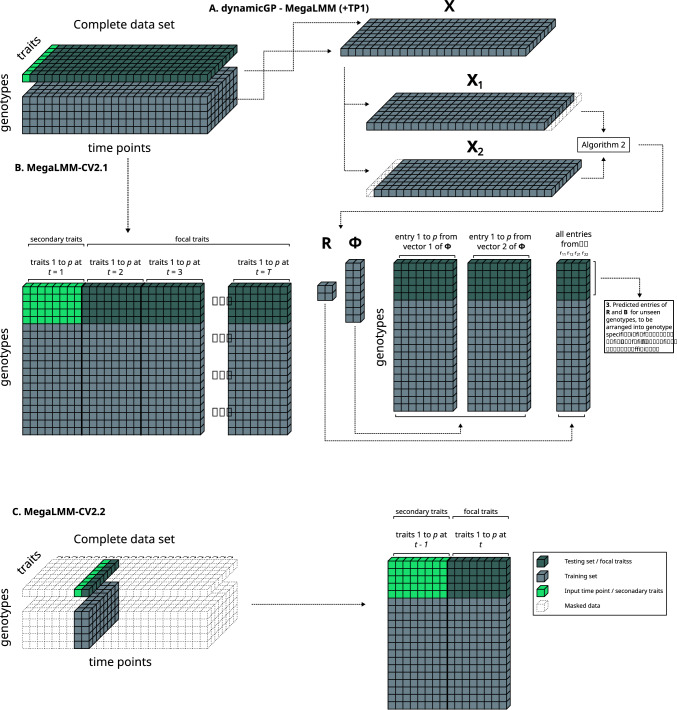
Illustration of the data usage in the compared MegaLMM formulations. **A** dynamicGP-MegaLMM (+TP1) In the first comparison, we used 80% of the data to train dynamicGP, MegaLMM and RR-BLUP models, and employed them to predict the remaining 20% of data. The recursive version of dynamicGP requires the initial (first) time point of the series as input. **B** MegaLMM-CV2.1 MegaLMM models were used to predict all traits and time points simultaneously employing the set of traits at the initial time point as secondary traits. **C** MegaLMM-CV2.2 MegaLMM models are trained and implemented using the set of traits from the focal time point as training data, along with the set of traits from the immediately preceding time point as secondary traits. This was repeated for each time point, for which a separate model was trained

## Materials and methods

### Data generation and pre-processing

A multi-parent advanced generation inter-cross (MAGIC) maize population of 347 recombinant inbred lines (RILs) and nine founders was phenotyped in three high-throughput experiments at the Leibniz Institute of Plant Genetics and Crop Plant Research in Gatersleben, Germany. Over 347,000 images were captured at multiple time points and processed to derive 498 geometric, color, and texture traits. A linear mixed model was used to estimate time-point-specific trait heritability and BLUP values as outlined in Hobby et al. (2025). Briefly, linear mixed models were fitted for each trait at each time point as

1 $$\begin{aligned} Y_{ijklm} = \mu + G_i + E_j + L_k + (G \times E)_{ij} + R(E)_{m(j)} + \epsilon _{ijklm}, \end{aligned}$$

where $G_i$, $E_j$, $L_k$, $(G \times E)_{ij}$, and $R(E)_{m(j)}$ are random effects for genotype, experiment, lane, genotype-by-experiment interaction, and replicate nested in experiment, respectively, and $\epsilon _{ijklm}$ is the residual error. This yielded a data set comprising BLUPs of 498 traits at 25 time points. The trait heritability was calculated as $h^2 = \frac{\sigma ^2_g}{\sigma ^2_t}$, where $\sigma ^2_g$ is the genotypic variance and $\sigma ^2_t$ is the total variance. One might expect that using the best linear unbiased estimates (BLUEs) instead of BLUPs may produce better results (Holland and Piepho 2024). However, we would like to keep the present study in line with our previous work (Hobby et al. 2025) to facilitate comparisons.

The BLUPs of these 498 traits were clustered based on the pairwise Mantel correlations between the genotype by time point matrices for each trait. Each trait was represented by a node in a network, with Mantel correlations above 0.95 as edge between two traits. Clusters of traits were found using modularity clustering as implemented in the R package “igraph” (Csardi and Nepusz 2006), from which the trait with the highest mean heritability across all time points was retained as representative, resulting in 50 selected traits from side, top and combined images. These traits were then min-max normalized and used for subsequent modeling (Hobby et al. 2025).

Genotyping data for 330 RILs with 79,557 single-nucleotide polymorphisms (SNPs) from SPET genotyping were used. Missing data were imputed using Beagle 5.2 (Browning et al. 2018), and the SNPs with a minor allele frequency of < 0.05 were filtered from the data. This yielded 70,846 SNPs which were then used in our genomic prediction models. The genomic relatedness matrix (GRM) required for MegaLMM was obtained using the kinship function in TASSEL 5 (Bradbury et al. 2007).

### DynamicGP implementation

Dynamic Mode Decomposition (DMD) determines a time-invariant best-fit linear operator matrix A that transforms a set of measurements, in our case a set of phenotypes, at one time point into the measurements of those same traits at the subsequent time point, i.e.,

#### Algorithm 1 (DMD)

2 $$\begin{aligned} {\textbf {x}}_{t+1} = {\textbf {A}} {\textbf {x}}_t \end{aligned}$$

where **x** represents a column vector of *p* traits and **A**, an *p* $\times$ *p* matrix. Concatenating the **x** vectors of *T* time points into matrices, Eq. (2) can be equivalently written as:

3 $$\begin{aligned} {\textbf {X}}_2 \approx {\textbf {A}} {\textbf {X}}_1 \end{aligned}$$

where **X**$_1$ and **X**$_2$ are *p* $\times$ (*T*-1) matrices offset by a single time point (Fig. 1A). From **X**$_1$ and **X**$_2$, the matrix **A** can be directly derived as:

4 $$\begin{aligned} {\textbf {A}} = {\textbf {X}}_2 {\textbf {X}}_1^\dagger \end{aligned}$$

where $^{\dagger }$ indicates the Moore–Penrose pseudoinverse. Algorithm 1, above, allows near-perfect recreation of the training data (Hobby et al. 2025); however, it is susceptible to noise and requires a large number of models (i.e., *p*$^{2}$ separate models for *p* traits) to be predicted if each matrix element is treated separately. Instead, dynamicGP uses an approximation algorithm termed Schur-DMD (Thitsa et al. 2021):

#### Algorithm 2 (Schur-DMD)

5 $$\begin{aligned}&{\textbf {X}}_1 = {\textbf {U}} \boldsymbol{\Sigma } {\textbf {V}}^\textrm{T} & \text {singular value decomposition of } {\textbf {X}}_1&\end{aligned}$$

6 $$\begin{aligned}&{ {\boldsymbol{\tilde{\mathrm A}}}} = {\textbf {U}}_{r}^{\rm{T}} {\textbf {X}}_2 {\textbf {V}}_r \boldsymbol{\Sigma }_{r}^{-1} & \text {rank-reduced POD-projected representation }\boldsymbol{ {{\tilde{\mathrm A}}}}&\end{aligned}$$

7 $$\begin{aligned}{}&\tilde{\boldsymbol{A}} = {\textbf {Q}}^\textrm{T} {\textbf {R}} {\textbf {Q}} & \text {Schur decomposition of }\tilde{\boldsymbol{{A}}}\end{aligned}$$

8 $$\begin{aligned}{}&\boldsymbol{\Phi } = {\textbf {X}}_2 {\textbf {V}}_r \boldsymbol{\Sigma }_r^{-1} {\textbf {Q}} & \text {projected DMD modes}&\end{aligned}$$

9 $$\begin{aligned}{}&{\textbf {A}}_r = \boldsymbol{\Phi } {\textbf {R}} \boldsymbol{\Phi }^\dagger & \text {reconstruction of approximated } {\textbf {A}}_r\end{aligned}$$

This approximation aims to preserve only the true signals within the data (i.e., noise filter). Further, it acts as a dimensionality reduction method, thereby reducing the number of matrix entries required to obtain a predicted **A** matrix from *p*$^{2}$ (i.e., Algorithm 1) to *r* $\times$ *p* for $\Phi$ and *r*$^{2}$ - z for **R** for *p* traits and *r* included singular values from the truncated model in Eq. (6). Here, *z* indicates the minimum number of zero entries in **R**, and is determined by the number of complex eigenvalues in $\mathbf {\tilde{A}}$; $$z = {\left\{ \begin{array}{ll} \dfrac{r (r-2)}{2} & \text {if } r \text { is even}, \\ \dfrac{(r-1)^2}{2} & \text {if } r \text { is odd} \end{array}\right. }$$.

The hyperparameter *r* was set to 2 based on a previous work (Hobby et al. 2025) where *r* was selected by estimating the heritability and prediction accuracy in cross-validation within the training set using RR-BLUP models of the elements within the intermediate matrices **U**, **V**, and $\boldsymbol{\Sigma }$, corresponding to their singular vector (Hobby et al. 2025), prior to validating model performance on the validation set.

The full set of traits at each time point were organized into genotype-specific trait $\times$ time point **X** matrices, which were then used as inputs to the Schur-DMD algorithm to find the intermediate component matrices **R** (*r* $\times$ *r*) and $\boldsymbol{\Phi }$ (*p* $\times$ *r*) (Algorithm 2; Fig. 1A). The matrix elements across multiple genotypes from these two matrices are treated as traits in genomic prediction models. DynamicGP initially used separate RR-BLUP models for each trait (denoted here as dynamicGP-RR-BLUP); here, we used multi-trait MegaLMM models to predict all the elements of the matrices simultaneously (dynamicGP-MegaLMM).

Between every fifth time point, there is a two-day gap which corresponds to a parallel phenotyping procedure which required the plants to sit in total darkness for 24 h. This precluded HTP RGB phenotyping for 48 h every 7 days. To account for this unequal gap in the time series we left out the time point immediately prior to the gap in **X**$_1$ and immediately following the gap in **X**$_2$, such that all corresponding pairs of columns in **X**$_1$ and **X**$_2$ represented a single day interval (Hobby et al. 2025).

Once predictions of the entries of **R** and $\boldsymbol{\Phi }$ were obtained for testing lines, they were organized into their corresponding matrices, and then used to derive genotype-specific time-invariant operator matrices **A**$_r$ using Eq. (9), which in turn allows for the prediction of traits at subsequent time points from the same traits at a given input time point. We performed 20 iterations of 5-fold cross-validation to predict the components of **R** and $\boldsymbol{\Phi }$, which were then employed in Eq. (9) to obtain predictions of **A**$_r$ for unseen genotypes (Fig. 1A).

### MegaLMM models

MegaLMM uses a two-level hierarchical model to predict multiple traits simultaneously. First, **Y** $\in \mathbb {R}^{(\textit{p} \times \textit{t})}$, a matrix of *p* genotypes and *t* traits, is decomposed into lower-dimensional *p* $\times$ *k* latent trait matrix **F** and *k* $\times$ *t* trait loading matrix $\boldsymbol{\Lambda }$, as well as an *p* $\times$ *t* matrix **E** of residuals:

10 $$\begin{aligned} {\textbf {Y}} = {\textbf {F}} \boldsymbol{\Lambda } + {\textbf {E}} \end{aligned}$$

Each of the *k* latent traits in **F** represents the variation of a subset of the original traits. The columns of **F** and **E** being linearly independent, can be modeled as independent linear mixed models of the following form:

11 $$\begin{aligned} {\textbf {M}} = {\textbf {X}} \boldsymbol{\beta } + {\textbf {Z}} \boldsymbol{\mu } + {\textbf {e}}. \end{aligned}$$

Here, **M** consists of **F** and **E** concatenated into a single matrix, **X** is an *p* $\times$ *f* matrix of fixed effects, $\boldsymbol{\beta }$ is a *f* $\times$ (*k* + *t*) vector of fixed effect sizes, **Z** is an *p* $\times$ (*k* + *t*) random effects design matrix, $\boldsymbol{\mu }$ is a *j* $\times$ 1 vector of random effect sizes, and **e** is an *p* $\times$ (*k* + *t*) matrix of residuals. By using this two-level approach, MegaLMM de-correlates the original data by isolating the underlying sources of variance and avoids performing repeated large matrix inversions typical of multi-variate linear mixed models. The number of factors *k* was selected using the recommended *k* = min(*p*/4, *t*/2) (Runcie et al. 2021).

#### Data usage

The present work applies MegaLMM in the following five ways:

1. **dynamicGP-MegaLMM**, where we substitute the RR-BLUP core of dynamicGP with MegaLMM to predict the building blocks of dynamicGP (**R** and $\boldsymbol{\Phi }$, Fig. 1A). All four entries of **R** were predicted in a single model, while the entries of the two vectors of $\boldsymbol{\Phi }$ were predicted separately and were then concatenated.
2. **dynamicGP-MegaLMM+TP1**, as above, the building blocks of dynamicGP were predicted with MegaLMM instead of RR-BLUP; however, in this configuration, all traits at the first time point were included as secondary traits to assist in predicting **R** and $\boldsymbol{\Phi }$. These traits are already visible to dynamicGP as the initial state.
3. **MegaLMM-CV1**, where we apply MegaLMM to the full set of 50 traits at all 25 time points in the given time series (Fig. S1A).
4. **MegaLMM-CV2.1**, where we apply MegaLMM using the full set of traits at the initial time point as secondary traits and predict the traits over the remaining 24 time points in the given time series—this represents the same data usage as recursive dynamicGP (Fig. 1B).
5. **MegaLMM-CV2.2**, where for all time points *t*, the set of traits at time *t*+1 are predicted using their available measurement at time *t* as secondary traits—serving as a comparison for the iterative version of dynamicGP, although it actually uses less data (Fig. 1C).

In methods 1–4 all traits at all time points are included in the training set. In methods 1, 2 and 4, all traits at the initial time point for the genotypes in the testing se are used in the model as either the initial state in dynamicGP, or secondary traits in MegaLMM-CV2.1, all traits at the remaining 24 time points comprise the testing set. For method 3, all traits at all 25 time points comprise the testing set. For method 5, the training set includes all traits at time *t* and *t*+1, while all traits at time *t* for the genotypes in the testing set are included as secondary traits to assist in predicting the testing set at time *t*+1. ST-STP RR-BLUP models for all trait-time-points and dynamicGP-RR-BLUP are included as reference points, (i.e., baseline models).

***Forecasting*** The three dynamicGP variants were further tested in a CV3 scenario (Momen et al. 2019; Hobby et al. 2025) in predicting unseen time points. In this scenario, phenotypic data from time points 1 to 20 were used for training, and time points 21 to 25 were used for testing. The phenotypic data for time point 20 was given as the initial state (Fig. S1B).

### Assessment of accuracy

Accuracy of time-series predictions was assessed in two different ways: (1) snapshot accuracy, for a trait and time point, Pearson’s correlation coefficient (PCC) between the true and predicted values of the trait across different genotypes was determined for each time point, resulting in separate prediction accuracies for each trait at each time point, and (2) longitudinal accuracy, for a trait and genotype, PCC and mean squared error (MSE) between the true and predicted values across time of the trait was determined for each genotype. We consider the longitudinal accuracy as the means to assess the accuracy of prediction of a trait’s dynamics. Snapshot accuracies are reported as aggregated accuracies across all traits and CV folds within a time point, while the longitudinal accuracies are aggregated across all traits and genotypes and CV folds. The accuracy of dynamicGP building blocks was assessed as the mean PCC between the true and predicted values across all CV folds. To test for significance of longitudinal predictions we determined the threshold PCC value as

12 $$\begin{aligned} \textrm{PCC}_{\text {threshold}} = \frac{t_{\text {crit}}}{\sqrt{t_{\text {crit}}^2 + n - 2}}, \end{aligned}$$

where $t_{\text {crit}}$ is the critical *t*-value determined from the *t*-distribution corresponding to the Bonferroni corrected *p*-value of 0.05, and *n* represents the number of elements in the tested vectors. Differences in accuracy between methods used to predict the building blocks of dynamicGP were assessed by performing pairwise t tests in R.

### Assessment of developmental trajectory characteristics

Several methods were used to quantify the characteristics (roughness and convexity) of the mean developmental trajectories of each trait **y**. To quantify roughness of the trajectory we used:

1. Total Variation: $$\text {TV} = \frac{1}{T-1} \sum _{i=1}^{T-1} |\Delta y_i|$$
2. Quadratic Variation: $$\text {QV} = \frac{1}{T-1} \sum _{i=1}^{T-1} (\Delta y_i)^2$$
3. Coefficient of Variation: $\text {CV} = \frac{\sigma _y}{\bar{y}} \cdot 100$
4. Root Mean Squared (RMS) roughness: $\text {Rq} = \sqrt{\frac{1}{T} \sum _{i=1}^T (y_{\text {detrended},i})^2}$
5. Kurtosis: $$\text {Rku} = \frac{\frac{1}{T} \sum _{i=1}^T (y_{\text {detrended},i})^4}{(\text {Rq})^4}$$

where the linear regression and de-trending are given by $\hat{\beta }_0, \hat{\beta }_1 = \text {argmin}_{\beta _0, \beta _1} \sum _{i=1}^n (y_{\text {norm},i} - (\beta _0 + \beta _1 x_i))^2, \quad y_{\text {detrended},i} = y_{\text {norm},i} - (\hat{\beta }_0 + \hat{\beta }_1 x_i), \quad \text {and} \quad \textbf{y}_{\text {norm}} = \frac{\textbf{y}}{\max (\textbf{y}) - \min (\textbf{y})}$.

We further quantified the convexity of the developmental trajectory using:

1. Mean Second Difference: $\Delta y_i = y_{i+1} - y_i$ $\Delta ^2 y_i = y_{i+2} - 2y_{i+1} + y_i$ $\text {MSD} = \frac{1}{T-2} \sum _{i=1}^{T-2} \Delta ^2 y_i$
2. Quadratic Coefficient: $$\hat{a}, \hat{b}, \hat{c} = \text {argmin}_{a, b, c} \sum _{i=1}^T (y_i - (a x_i^2 + b x_i + c))^2$$ $$\text {QC} = \hat{a}$$
3. Mean Chord Deviation: $\text {chord}_{i,j,k} = \frac{(k - j) y_i + (j - i) y_k}{k - i}$ $\text {dev}_{i,j,k} = \text {chord}_{i,j,k} - y_j$ $\text {MCD} = \frac{1}{|\mathcal {T}|} \sum _{(i,j,k) \in \mathcal {T}} \text {dev}_{i,j,k}$ where $\mathcal {T} = \{(i,j,k) \mid 1 \le i< j < k \le T\}$

## Results

### Set-up for comparative analysis of dynamicGP and MegaLMM

The comparative analysis between dynamicGP and MegaLMM utilized 50 geometric, color, and texture traits measured across 25 time points in 330 maize lines from a MAGIC maize population, resulting in 1250 unique trait-time-point pairs (see Materials and Methods). Five distinct MegaLMM approaches were implemented. In the first approach, termed dynamicGP-MegaLMM, single-trait RR-BLUP models in dynamicGP were replaced with multi-trait MegaLMM models. Here, the four elements of the **R** matrix (*r*$^{2}$=4) were predicted simultaneously using a single multi-trait model, while the two vectors of $\boldsymbol{\Phi }$ (*r*=2), each containing 50 elements corresponding to the 50 traits (Eq. (8), Schur-DMD), were predicted individually using two separate multi-trait models. These predictions were then organized into their respective matrices and integrated into the dynamicGP algorithm (Eq. (9), Materials and Methods), requiring three MegaLMM models per cross-validation iteration. Once genotype-specific **A**$_r$ matrices are calculated they can be employed in iterative or recursive fashion.

The second approach extends the first by considering the initial phenotypic state included as secondary traits to assist in predicting matrix elements. The initial state is visible to dynamicGP models already, so this does not introduce data leakage. In the third approach, the full time series comprising all 1250 trait-time-point pairs were predicted together in a single MegaLMM model and compared to univariate RR-BLUP models for each trait-time-point pair as a reference point. In the fourth approach, the MegaLMM-CV2.1 “recursive” scenario, a single MegaLMM model was trained using all 50 traits at *t*=1 as secondary traits to predict these traits across time points 2 to 25, yielding predictions for 1200 trait-time-point pairs (Fig. 1B). This matches the data usage for the recursive configuration of dynamicGP. In the fifth approach, the MegaLMM-CV2.2 “iterative” scenario, multi-trait MegaLMM models were trained iteratively, using all traits at one time point (e.g., *t*) as secondary traits to predict traits at the next time point (*t*+1), repeating this process across the time series from *t*=1 to *T*-1 (Fig. 1C), requiring a separate model for each time point.

### MegaLMM more accurately predicts building blocks of dynamicGP

**Fig. 2:**
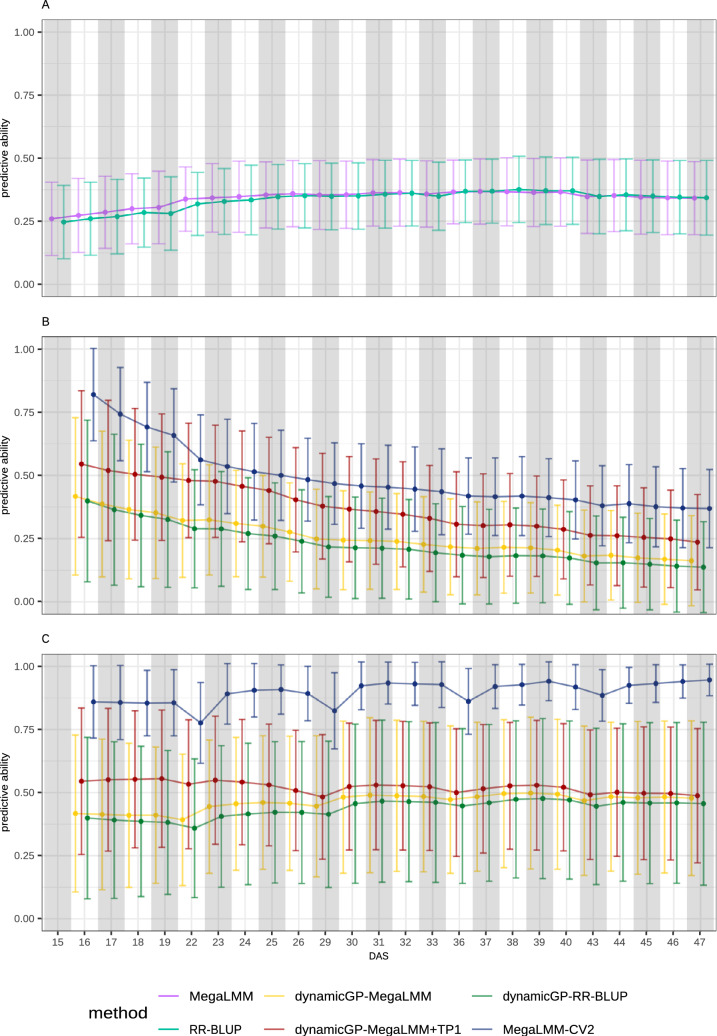
MegaLMM with secondary traits outperforms dynamicGP in terms of snapshot accuracy.** A** Mean time-point-specific snapshot accuracy across all traits for RR-BLUP models in ST-STP configuration and MegaLMM models in MT-MTP configuration with all traits and time points simultaneously with no secondary traits.** B** &** C** Mean time-point-specific snapshot accuracy for dynamicGP models using either RR-BLUP or MegaLMM as core method for $\mathbf {\Phi }$ and **R** prediction. DynamicGP-MegaLMM was tested both with (+TP1) and without the initial phenotypic state included as secondary traits. **B.** Recursive configuration and MegaLMM-CV2.1 with the initial phenotypic state as secondary traits to predict the remainder of the time series. **C.** Iterative configuration with MegaLMM-CV2.2 with the phenotypic state at a single time point as secondary traits to predict the following time point

**Fig. 3:**
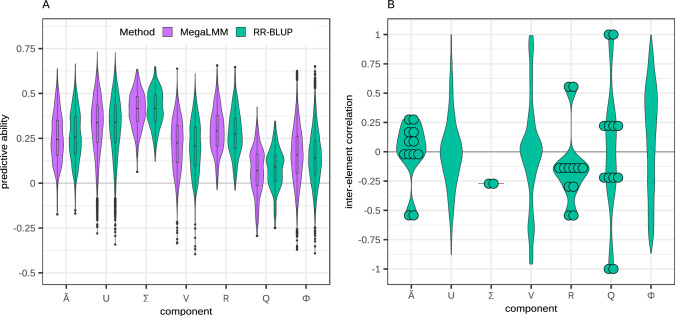
MegaLMM yields higher predictive ability for some of the building blocks of dynamicGP. **A** Distribution of predictive abilities for the elements of intermediate matrices used in dynamicGP using MegaLMM and RR-BLUP models. **B** Distributions of inter-element correlation within the intermediate matrices used in dynamicGP. Circles represent inter-element correlations for matrices with four or fewer elements

Here, we compared the accuracy with which univariate RR-BLUP and multi-variate MegaLMM models were able to predict the building blocks of dynamicGP. To this end, we applied dynamicGP to multi-trait time-series data comprising 50 traits measured across 25 time points. Individual models were constructed to predict the elements of the **R** and $\boldsymbol{\Phi }$ matrices, which were then arranged into their corresponding matrices to calculate genotype-specific **A**$_r$ matrices for longitudinal predictions (see Materials and Methods). While the original dynamicGP implementation utilized *r* $\times$ *n* + *r*$^{2}$ RR-BLUP models, these were replaced with three MegaLMM models in the present study (see Materials and Methods). Using MegaLMM to predict the **R** and $\boldsymbol{\Phi }$ components, which were subsequently integrated into dynamicGP, resulted in an increase in mean predictive ability of 8% across all time points, representing an increase in the recursive and iterative configurations of 11% and 6%, respectively (Fig. 2). The inclusion of the initial phenotypic state yielded predictions 21% higher than the RR-BLUP-based approach, corresponding to 53% and 18% increases for the recursive and iterative configurations, respectively. Further analysis of the predictive ability of the building blocks of dynamicGP revealed that MegaLMM models yielded 3% higher (*p*-value = 4.28E-06) mean accuracies across all matrices and elements after 20 iterations of 5-fold cross-validation (Fig. 3A), although not all matrices nor all elements were better predicted by MegaLMM when examined individually. For example, **Q** and some elements from the other matrices were better predicted by RR-BLUP, while for others, no difference was observed/noticeable between the two methods (Table S3). When the initial time point was included as secondary traits when predicting the building blocks, the prediction accuracy for the **R** elements increased by 75% and 65% compared to RR-BLUP and MegaLMM, respectively, and for the elements of $\boldsymbol{\Phi }$ the increase was 54% and 36%, respectively (Fig. S5). In addition, the inter-element correlations in dynamicGP components were assessed, showing a mean correlation near 0 for most components, with nonzero mean correlations observed for $\boldsymbol{\Sigma }$, **R**, and $\boldsymbol{\Phi }$ (Fig. 3B).

### MegaLMM outperforms dynamicGP with matching data usage

Predicting all time points together in a single multi-variate MegaLMM model yielded a predictive ability increase of 2% over the univariate ST-STP RR-BLUP models (Fig. 2A). We note a tendency within both methods for increasing accuracy from the initial time point until 36 days after sowing, after which predictive ability stabilized until 40 days after sowing. MegaLMM performed better in the early time points, while RR-BLUP performed better than MegaLMM in the later time points (Fig. 2A). No differences between the two methods were observed in terms of MSE (Fig. S6A).

MegaLMM was applied directly to the time-series data in two further configurations in addition to its use within dynamicGP. In the first configuration (complete time-series prediction), the initial phenotypic state, i.e., 50 traits at *t*=1, were used as secondary traits to predict the remaining 1200 unique trait-time-point pairs (50 traits across 24 time points) from genomic data, aligning with the data usage of recursive dynamicGP. In the second configuration (iterative time-series prediction), 50 traits at a given time point (e.g., *t*=1) were used as secondary traits to predict the same traits at the next time point (e.g., *t*=2), with this process repeated sequentially using separate models for each transition (e.g., *t*=2 to *t*=3), utilizing less data than iterative dynamicGP by including only the focal and immediately preceding time points.

For the complete time-series configuration, MegaLMM-CV2.1, serving as a comparison for the recursive configuration of dynamicGP, outperformed the best performing version of dynamicGP, dynamicGP-MegaLMM+TP1, by approximately 24% in snapshot accuracy across the full time series (Fig. 2B). Predictive abilities decayed over time for all versions of dynamicGP and MegaLMM-CV2.1 (Fig. 2B). MegaLMM-CV2.2, serving as a comparison for the iterative configuration of dynamicGP, outperformed iterative dynamicGP-MegaLMM+TP1 by approximately 66% across the time series (Fig. 2C), achieving a mean prediction accuracy of 0.90 ± 0.12 compared to 0.54 ± 0.27 for dynamicGP-MegaLMM+TP1. In this configuration, prediction accuracy dipped noticeably at every fifth time point corresponding to a two-day gap in the time series every five days, and a slight increase in accuracy was observed toward the end of the time series for dynamicGP-RR-BLUP, dynamicGP-MegaLMM, and MegaLMM-CV2.2 (Fig. 2C). Interestingly, although the MegaLMM-CV2 approaches outperformed dynamicGP in both recursive and iterative configurations, the MegaLMM-based methods had higher snapshot MSEs than all versions of dynamicGP at all time points (Fig. S6B and C). The inclusion of the initial time point as secondary traits in MegaLMM-CV2.1 yielded a more than 200% increase in mean prediction accuracy across all traits and time points compared to MegaLMM-CV1. This is largely due to the very high accuracies observed in the first time points directly after the initial time point, while the accuracies at the later time points are more similar between the two MegaLMM variants.

### Classical GP methods more accurately reproduce trait trajectories

To assess the ability of dynamicGP and MegaLMM to predict trait dynamics beyond snapshot accuracies, longitudinal prediction accuracy was evaluated using the Pearson correlation and mean squared error between predicted and true trait values across time points for each trait and genotype, across 20 iterations of 5-fold cross-validation.

**Fig. 4:**
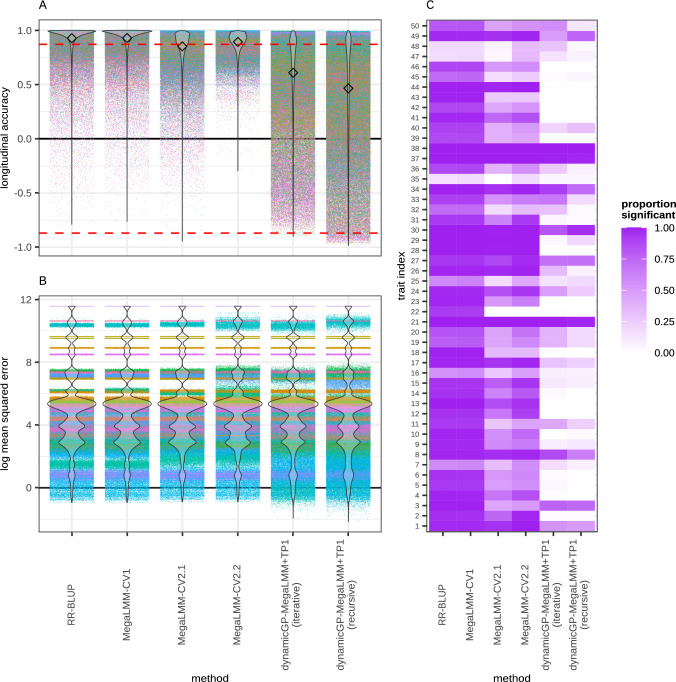
Classical GP models have highest longitudinal accuracy and proportion of significant correlations. **A** Accuracy of predicted trait dynamics along time series aggregated across all iterations, traits, and genotypes for univariate RR-BLUP models for each trait-time-point pair, MegaLMM-CV1, MegaLMM-CV2.1 and MegaLMM-CV2.2 as well as both iterative and recursive variants of dynamicGP-MegaLMM+TP1. Accuracy was assessed as the Pearson correlation between true and predicted values. All values above the red dashed lined are significant with Bonferroni adjusted *p*-values $\le$ 0.05. Diamonds indicate the mean prediction accuracy for each method. **B** Log transformed mean squared error of predicted trait dynamics along time series aggregated across all iterations, traits, and genotypes for the six methods. Within each method, there are a total of 330,000 tests performed, corresponding to the total number of combinations of 50 traits over 330 genotypes and 20 cross-validation, with each indicated by a point. Colors in A. & B. indicate different traits. **C** Proportion of significant positive predictive correlations for traits using each of the six methods. For an extended figure containing the corresponding results for the other methods please see Figures S2, S3 and S4. Supplementary Table 1 contains mapping of traits to index

The classical GP methods RR-BLUP and MegaLMM performed similarly both exhibited the highest mean PCC of 0.92 ± 0.13 across all 50 traits (Fig. 4A). These values were obtained by omitting predictions of the initial time point, in order to make a fair comparison with the other methods which did not make predictions of the initial time point. When the initial time point was included in the assessment, the accuracy increased for both methods to 0.93 ± 0.13 (Fig. S2). The two CV2 MegaLMM methods also performed well in terms of PCC, with MegaLMM-CV2.2 having a mean of 0.85 ± 0.16, and MegaLMM-CV2.2 with a mean of 0.89 ± 0.08 (Fig. 4A). The three variants of dynamicGP—RR-BLUP, MegaLMM, and MegaLMM+TP1—all performed similarly within the recursive and iterative configurations (Fig. S2A), with the dynamicGP-MegaLMM+TP1 performing slightly better on average between the two configurations than the RR-BLUP and MegaLMM-based versions, with mean PCCs of 0.46 ± 0.47 and 0.61 ± 0.39 for iterative and recursive, respectively (Fig. 4A). DynamicGP-MegaLMM performed best in the recursive configuration with a mean PCC of 0.47 ± 0.47 (Fig. S2A).

We found that all tested methods displayed similar distributions of MSE values across all traits, genotypes, and cross-validation iterations and folds. Moreover, similar banding patterns for individual traits could be observed within the MSE distributions for each method (Fig. 4B and S2B). Similarities were especially pronounced between ST-STP RR-BLUP and MegaLMM-CV1, as well as within the two configurations of dynamicGP. The iterative configuration of dynamicGP performed best with mean MSEs of 4542.27, 4544.98, and 4545.00 observed for dynamicGP-MegaLMM+TP1, -MegaLMM, -RR-BLUP, respectively. The two classical GP methods ST-STP RR-BLUP and MegaLMM-CV1 also performed similarly, with mean MSEs of 4588.70, and 4589.72, respectively. Mean MSE for these methods decreased to 4578.54 and 4579.72, respectively, when the first time point was also considered (Fig. S2B). MegaLMM-CV2.1 outperformed MegaLMM-CV2.2 with a mean MSE of 4602.61 versus 4616.68. The three versions of the recursive configuration of dynamicGP had the highest overall mean MSEs with 4673.24, 4682.2, and 4684.70 for dynamicGP-MegaLMM+TP1, -MegaLMM, -RR-BLUP, respectively. Untransformed mean MSEs are presented here to avoid biasing the presentation of the results, although they are plotted in log-transformed axis to assist in visualization (Fig. 4B and S2B).

The two classical GP methods ST-STP RR-BLUP and MegaLMM-CV1 exhibited the highest percentage of significant correlations, with both having 84% of all correlations significant after Bonferroni multiple testing correction. The majority of traits showed high proportion of significant correlations for these two methods (Fig. 4C). The trait-wise proportions were relatively unchanged irrespective of whether or not the first time point was considered (Fig. S3 and S4), although the overall percentages increased to 86% and 87% for ST-STP RR-BLUP and MegaLMM-CV1, respectively. MegaLMM-CV2.1 and -CV2.2 resulted in 55% and 66% significant correlations, respectively. Both MegaLMM-CV2 methods showed high proportion of significant correlations, with MegaLMM-CV2.2 as a dominant method (Fig. 4C). The iterative dynamicGP methods results in 26% significant correlations, while recursive dynamicGP resulted in 20–21% significant correlations. Both configurations of dynamicGP showed low numbers of traits with high proportions of significant correlations (Fig. 4C), with no major differences between the different versions of dynamicGP (Fig. S3 and S4). The significant correlations yielded by ST-STP RR-BLUP and MegaLMM models were almost all positive correlations, with only a single trait having significant negative correlations for MegaLMM-CV2.1 (Fig. S4). In contrast, all versions and configurations of dynamicGP yielded multiple significant negative correlations (Fig. S4). We note that the traits with low proportion of significant correlations with MegaLMM-CV2.1 and -CV2.2 correspond to those traits in which the observed mean trajectory exhibited a large degree of “jaggedness” (i.e., many abrupt changes in direction). Given that the MegaLMM-CV2 models closely match the observed trajectories, only with a one time point delay, similar trajectories with large changes in direction when offset by a single time point will exhibit larger differences between predicted and observed trajectories than a smooth trajectory. This observation is further substantiated by the finding of weak negative correlations between both the mean longitudinal PCC and proportion of significant correlations for a given trait using the MegaLMM-CV2 methods and common measures of the “roughness” of a function (total variation (−0.18 to −0.16), quadratic (−0.16 to −0.14) variation and RMS roughness (−0.27 to −0.19) applied to the mean observed trajectory, although none of these correlations are significant (Table S2). Similarly, the traits with a high proportion of significant correlations from dynamicGP tended to be the traits which had trajectories which were either consistently increasing or decreasing or contained only sweeping smooth fluctuations. Convex and concave trajectories and those with many jagged points, judged by visual analysis, tended to be poorly reproduced by dynamicGP. When we quantified these properties, we only observed that the mean longitudinal PCC and proportion of significant correlations from iterative dynamicGP displayed weaker correlations against RMS roughness and stronger negative correlations against mean second difference of the mean observed trajectory compared to the other tested methods (Table S2). Against other measures of convexity—quadratic coefficient and mean chord deviation—mean longitudinal PCCs from dynamicGP displayed comparatively weaker negative (not significant) correlations than those from the other tested methods (Table S2).

**Fig. 5:**
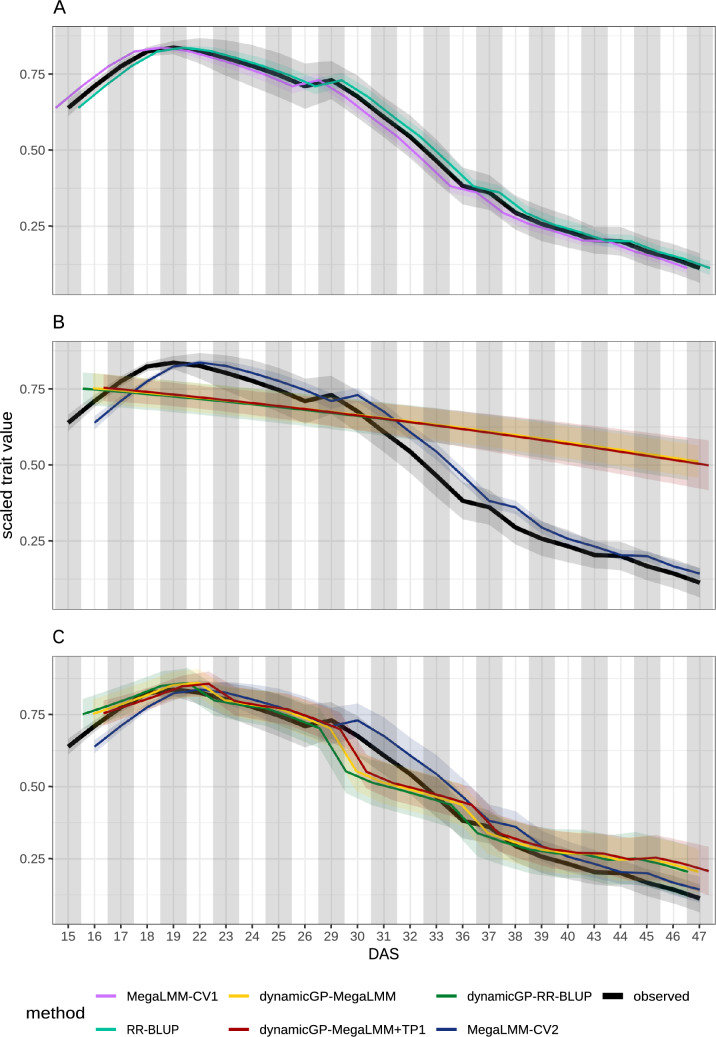
Comparative analysis of MegaLMM and dynamicGP variants based on predicted developmental trajectories. Predicted trajectory of the trait with the highest mean longitudinal accuracy (*top.intensity.vis.lab.a.skewness*) across all of the tested methods using (**A**) ST-STP RR-BLUP models, and MegaLMM-CV1 models; three versions of the (**B**) recursive configuration of dynamicGP (RR-BLUP, MegaLMM, MegaLMM+TP1), and MegaLMM-CV2.1; and (**C**) corresponding iterative versions of dynamicGP and MegaLMM-CV2.2. Predicted trajectory is the mean predicted value of a given trait at each time point across all cross-validation iterations and folds, shading represents standard deviation

When it comes to reproducing the actual developmental trajectories the CV1 approaches performed best, with ST-STP RR-BLUP and MegaLMM-CV1 producing curves which not only displayed high accuracy in terms of PCC between predicted and observed trajectory, but closely matched the time temporal fluctuations (Fig. 5A and S7A). MegaLMM-CV2.1 and -CV2.2 similarly reproduce curves which closely match the temporal fluctuations, albeit with a single time point delay (Fig. 5B, C, S7B and C). The recursive dynamicGP was the worst performing methods for developmental trajectory prediction, with the three variations performing similarly and resulting in smooth curves with no sharp changes which do not follow the trends in the observed curves (Fig. 5B and S7B). The iterative configuration of dynamicGP yielded trajectory predictions which tended to follow the broad trends in the observed trajectory but did not match the finer temporal fluctuations (Fig. 5C).

### DynamicGP remains the only approach which can forecast multiple traits simultaneous

Although the MegaLMM methods and ST-STP RR-BLUP models outperformed the dynamicGP methods in terms of both snapshot and longitudinal accuracies, these accuracies were assessed within the set of training time points. However, when it comes to forecasting, (i.e., predicting time points which come after the time points in the training set) the static GP methods are not applicable. In terms of snapshot accuracy it may be fair to assume correlation with the final time point within the training set for a number of time points but this would result in the same predictions for all forecasted time points, rendering assessment for longitudinal accuracy impossible due to the lack of variance.

In order to demonstrate this ability of dynamicGP we trained models for each version of dynamicGP on data from the first 20 time points, and then used these models to forecast the final 5 time points. We again assessed the accuracy of these predictions in terms of PCC and MSE in both snapshot and longitudinal formats.

In forecasting mode, dynamicGP-MegaLMM+TP1 was again the best performing variant, with mean snapshot accuracies of 0.56 and 0.57, for recursive and iterative configurations, respectively, while dynamicGP-MegaLMM exhibited the lowest snapshot PCCs with 0.51 and 0.53, respectively (Fig. S8). DynamicGP-RR-BLUP yielded intermediate snapshot PCCs of 0.53 and 0.55 for recursive and iterative, respectively. Further, when MSE was used as the evaluation metric and despite its tendency to increase from early to later time points (Fig. S9), no large differences were observed between variants of the models evaluated. In terms of longitudinal PCC, all dynamicGP variants performed relatively poorly, with mean accuracies of  0.06 and 0.15 for both the -RR-BLUP and -MegaLMM variants in iterative and recursive configurations, respectively. The -MegaLMM+TP1 variant had both the highest mean PCC in the iterative configuration with 0.07 and the lowest mean PCC in the recursive configuration with 0.13 (Fig. S10A). None of the correlations proved to be significant. Although the mean longitudinal PCCs of the iterative and recursive configurations are not very different, the distributions of accuracies across traits and genotypes differed. The iterative configuration yielded a normal distribution ranging from −1 to 1, while the recursive configuration yielded a bimodal distribution with peaks near 1 and −1 (Fig. S10A). In terms of longitudinal MSE all methods performed similarly (Fig. S10B).

## Discussion

The four MegaLMM implementations provided distinct frameworks for comparison with dynamicGP-RR-BLUP, each providing different means for using genomic data. The dynamicGP-MegaLMM, and -MegaLMM+TP1 approaches extended the original dynamicGP by incorporating MVGP to predict **R** and $\boldsymbol{\Phi }$ components, enabling simultaneous modeling of multiple elements. This hybrid method contrasts with the MegaLMM-CV2.1 “recursive” scenario, which utilized a single model to predict 1200 trait-time-point pairs based on genomic data along with initial time point data (*t*=1), offering a comparison to the recursive dynamicGP-RR-BLUP setup. In contrast, the MegaLMM-CV2.2 “iterative” scenario used step-wise prediction, training separate models for each time point, which mirrors the iterative dynamicGP-RR-BLUP configuration, but takes advantage of inter-trait correlations within and between individual time points. Although less data actually enter the model compared to dynamicGP, MegaLMM-CV2.2 makes use of only the focal time point with the preceding time point as secondary traits, while dynamicGP employs the entire time series as training data with a single time point given to predict the following time point. Our findings showed that MegaLMM-CV2.2 outperformed the corresponding dynamicGP models considerably in terms of both snapshot and longitudinal accuracies. The MegaLMM-CV2.1 approach stands out for its ability to handle the full time series in a single model, potentially reducing computational overhead compared to the MegaLMM-CV2.2 approach, which requires 24 distinct models for the 25 time points. These configurations highlighted trade-offs between model complexity, predictive scope, and alignment with dynamicGP-RR-BLUP’s recursive and iterative frameworks, providing a basis for evaluating their performance against dynamicGP-RR-BLUP (see Materials and Methods). The MegaLMM-CV1 and ST-STP RR-BLUP implementations offer a benchmark against which we can determine the benefit of including the initial time point in MegaLMM longitudinal models.

The replacement of RR-BLUP models with three MegaLMM models in dynamicGP-MegaLMM yielded an increase in mean prediction accuracy of 8% across all time points in both recursive and iterative configurations (Fig. 2B and C). This increase is up to 30% when the initial time points are included in the MegaLMM models for predicting **R** and $\boldsymbol{\Phi }$. This finding indicates that MegaLMM as employed here is a suitable replacement for RR-BLUP within dynamicGP. This increase in performance prompted an investigation into MegaLMM’s prediction accuracy of the elements of the intermediate matrices **R** and $\boldsymbol{\Phi }$ as well as the other matrices from the Schur-DMD algorithm, which revealed generally similar accuracies, although **R** and $\boldsymbol{\Phi }$ showed slightly higher accuracies with MegaLMM and especially MegaLMM+TP1 (Fig. 3A and S5). Given that MegaLMM exploits inter-trait covariance to enhance prediction accuracy over single-trait methods, the near-zero mean correlations among the elements of most dynamicGP intermediate matrices (Fig. 3B) likely undermined its effectiveness. Nonzero mean correlations were observed for $\mathbf {\tilde{A}}$, $\boldsymbol{\Sigma }$, **R**, and $\boldsymbol{\Phi }$ (Fig. 3B), only **R**, and $\boldsymbol{\Phi }$ showed a predictive advantage.

The direct application of MegaLMM to time-series data revealed distinct performance advantages over all versions of dynamicGP in both configurations. Using MegaLMM-CV2.1 “recursive” models, we found a 24% improvement in accuracy across all traits and time points over the best performing dynamicGP version, dynamicGP-MegaLMM+TP1 (Fig. 2B). This demonstrates its ability to extract meaningful information from the initial trait data (*t*=1), used as secondary traits, for robust long-term predictions, unlike recursive dynamicGP, where error propagation, accumulating from each prediction step, likely drove the accuracy decline (from 0.54 to 0.23). The decay in accuracy for MegaLMM-CV2.1 (0.82–0.37) may stem from decreasing trait autocorrelation and genetic correlation as temporal distance increases, reducing the predictive power of early time points compared to later ones. This observation is concordant with the results of Momen et al. (2019), who used random regression models trained on early time points to predict later ones and observed a decay in accuracy in the later time points, although the cross-validation scenario used corresponded to CV3 as implemented here. The gain in accuracy of the MegaLMM-CV2.2 “iterative” model over iterative dynamicGP-MegaLMM+TP1 (mean 0.90 vs. 0.54) reflects its effective use of pairwise time-point data, although it uses less data than iterative dynamicGP, suggesting that immediate trait relationships are sufficient for accurate predictions (Fig. 2C). It is important to note in the comparison of dynamicGP to either of the MegaLMM-CV2 models that due to the truncation step within dynamicGP, there is an approximately 20% loss of variance in the data before any predictions are performed, while there is no equivalent step within MegaLMM. The observed accuracy of predictions from all CV2 models dips at every fifth time point, aligning well with a two-day measurement gap after each five-day interval, causing weakened inter-time-point trait autocorrelations compared to the 24-hour gaps between all other sequential time points; this observation in part explains the periodic reductions in accuracy. This gap is due to a parallel phenotyping procedure conducted during these growth experiments in which the plants were required to sit in total darkness for 24 h prior to being measured for chlorophyll fluorescence traits, which precluded the RGB measurements for 48 h every 7 days. The slight accuracy increase late in the time series could indicate that genetic differences become more strongly expressed in mature plants, enhancing MegaLMM’s ability to distinguish trait variations compared to earlier stages. This observation, although less pronounced and with lower overall mean accuracy, is in line with previous studies using random regression models in the CV1 scenario (Baba et al. 2020; Momen et al. 2019). These results suggested the superiority of MegaLMM in direct time-series applications, particularly when error propagation or data gaps impact the performance of dynamicGP.

It is interesting to point out the following important properties with respect to some matrices derived from DMD and subsequently used in the prediction tasks. (i) The matrix **R** is both quasi-triangular and block diagonal, and as such very likely it contains zero entries as well as identical entries along the diagonal. The implication of this property is the existence of non-unique $r^{2}$ entries in **R**. Even if in the current analysis no zero entries were found in **R** (using $r=2$), this is something that should be considered for effective model building in future studies. (ii) Many, but not all, genotype-specific **R** matrices can have identical diagonal entries as was the case with the investigated data set. This indicates the existence of complex eigenvalues for the corresponding genotype-specific $\mathbf {\tilde{A}}$ matrices. (iii) Because a constant (across all genotypes) phenotypic vector cannot be used for GP, a sanity check should always be performed on the vectors corresponding to the diagonal entries of **R** to see if they are identical or not. In the current analysis, we observed the presence of identical entries for some genotype but the resulting pseudo-traits at the population level had distinct entries and, therefore, were treated as independent traits. (iv) For real-valued input, it is known that the eigenvalues occur in complex-conjugate pairs (Krake et al. 2021). This means that in terms of reconstruction, their imaginary contributions will cancel out, leading to the reconstructed output that is real-valued. The presence of these complex-conjugate eigenvalues is related to the dependence of entries along the diagonal of **R**. However, we did not consider the lack of independence between complex eigenvalues, here present in about $50\%$ of the investigated genotypes. It would be interesting to explore such behavior in future research and see to what extent it can affect computational complexity and model performance.

While snapshot accuracies provide a basis for ranking and selecting genotypes within a single time point, they do not capture the ability to predict temporal trait trajectories, as high PCCs at individual time points may not accurately reflect the shape of the trait trajectories. Longitudinal analysis revealed that the static GP models outperformed all versions of dynamicGP in developmental trajectory recreation, suggesting better accuracy in tracking trait dynamics over time (Fig. 5 and S7). Interestingly, similar methods (i.e., MegaLMM-CV1 and ST-STP RR-BLUP; all versions of dynamicGP; and MegaLMM-CV2.1 and -CV2.2) displayed similar longitudinal PCCs and MSEs, despite the fact that they differed in terms of snapshot PCC and MSE (Fig. 4 and S2). The shapes of the predicted curves were likewise very similar among similar methods (Fig. 5 and S7). We find the observation that static approaches better reproduce developmental trajectories unsurprising, because in order to match a trajectory over time, a model simply needs to produce predictions within the same range of values as the observed values, which is a trivial task with correctly implemented scaling. In contrast, modeling a trait’s trajectory in a dynamic model, requires an understanding of how the curve actually unfolds across time, and how a value at a given time point influences the value in the following and subsequent time points, as we observe with iterative dynamicGP (Fig. 5 and S7). We hypothesize that the observation that predicted trajectories from iterative dynamicGP match the general tendencies within a curve, while missing the exact temporal fluctuations is likely due to a combination of the truncation step, which removes  20% of the variance in the data, as well as the accrued error stemming from the genomic prediction step, which yields imperfect predictions of **R** and $\boldsymbol{\Phi }$, leading to imperfect predictions of **A**$_r$. Further, the observation that recursive dynamicGP produces extremely smooth curves which fail to reproduce the broad scale dynamics of a trait’s trajectory suggest that it is not suitable for trajectory modeling on its own. Future studies could focus on the role of truncation and prediction error in developmental trajectory prediction using dynamicGP. To our knowledge no other studies to date have examined the performance of static or dynamic GP methods in recreating the actual developmental curves, nor have examined longitudinal accuracy via PCC or MSE.

Trait-specific analysis revealed that the superior performance of the static models carries over to a high proportion of significant longitudinal predictive correlations for the majority traits. Interestingly the inclusion of secondary traits in the static models (MegaLMM-CV2.1 and -CV2.2 vs. MegaLMM-CV1 and ST-STP RR-BLUP) actually decreased the proportion of significant correlations in general. Through our visual and quantitative analysis of the characteristics of the mean developmental trajectories, we found that the RMS roughness, (i.e., high variance between neighboring time points) of a trait’s trajectory exhibited a weak negative association to both mean longitudinal accuracy and to the proportion of significant correlations for MegaLMM-CV2.1 and -CV2.2 (Table S2). Though these correlations were not significant they may point in a direction for future analysis. It is surprising that although visual analysis of the observed trait trajectories alongside the predicted trajectories from dynamicGP suggested that the roughness and convexity of a trajectory would be negatively correlated with both longitudinal accuracy and proportion of significant correlations, we were unable to demonstrate this through any of the tested quantification approaches, and in fact may have found weak evidence to the contrary, in that longitudinal accuracy and proportion of significant correlations from iterative dynamicGP models were less associated with RMS roughness than the corresponding accuracies from other tested approaches (Table S2). Similarly, although both longitudinal accuracy and proportion of significant correlations from iterative dynamicGP models showed very weak negative associations with one convexity quantification approach (MSD), which were slightly higher than for other tested methods, we found the opposite trend for both iterative and recursive dynamicGP for the other two tested convexity quantification approaches (QC and MCD) (Table S2). Again, although these correlations did not prove to be significant, they may point in a direction for future analysis. One weak point in this analysis is that we conducted the roughness and convexity quantification on the mean trait trajectory, rather than on the individual trajectories for each genotype. We also treated all inter-time-point variance within the time series as equally important. It seems logical that the point in the time series where high inter-time-point variance is located would be relevant, particularly for recursive dynamicGP.

The snapshot accuracies attained here in forecast with dynamicGP were considerably lower on average when compared to other studies which implemented dynamic MVGP methods. For instance, Baba et al. (2020) achieved a mean predictive correlation above 0.8 for water usage with and without projected shoot area (PSA) as a secondary trait using multi-variate RRMs trained on the first 10 days of a time series and used to predict the following 10 days. However, these comparisons are either not strictly forecasting, or not multi-trait. When comparing to univariate forecasting approaches dynamicGP as implemented here is also less accurate than RRMs were for forecasting PSA at later time points based on earlier measurements (Momen et al. 2019).

Although the MegaLMM-based approaches outperformed dynamicGP in the present work, dynamicGP still has a optimistic outlook for two reasons. It is important to keep in mind that the time series used in the present study is still relatively small, comprising only 50 traits and only 25 time points at a one day time interval measured under stable environmental conditions. The resulting genotype $\times$ (trait $\times$ time point) matrix is a still manageable size (1200 or 1250 columns) for MegaLMM. With expansion of HTP technologies, it is conceivable if not likely that in the near future there will be data sets available comprising hundreds or thousands of traits and with much shorter time intervals, potentially as low as every few minutes, resulting in huge numbers of time points. Can MegaLMM handle a time series comprising 1000 traits with measurements every 5 min for 3 months? The resulting genotype $\times$ (trait $\times$ time point) matrix in this case would have 25.9 million columns. At this scale, it is likely that MegaLMM-based approaches will become computationally infeasible, while the dynamicGP equivalent would only need a few thousand predicted matrix elements. This would leave dynamicGP as the sole longitudinal MVGP approach capable of making use of data at such scale. The second reason for optimism for dynamicGP is its ability to perform forecasting. As a static method, MegaLMM is unable to predict the actual dynamics of a developmental trajectory, while dynamicGP, however imperfectly, does this implicitly.

With static MVGP models agronomically relevant traits, such as end of season yield, which are measured at a single time point, can simply be concatenated to the matrix containing each trait and each time point; however, it is unclear at this stage how can be integrated into the dynamicGP framework. This is a point for potential future research.

DynamicGP, in its current form, requires equidistant time points. In the present study, the data contained a two-day gap every seven days. In order to use this data within dynamicGP, we had to drop the vectors immediately preceding and following the gaps in **X**$_1$ and **X**$_2$, such that the paired columns in the two matrices corresponded to a one-day gap. There are extensions to DMD which can function with variable spacing (Askham and Kutz 2018; Li et al. 2022), which may prove useful in future developments of dynamicGP, especially in light of the tendency for field-collected data sets to have uneven spacing throughout the growing season. Additionally, different phenotyping technologies work on different time scales, potentially leading to data sets with traits measured at different resolutions. In order to make use of this data within the current formulation of dynamicGP, the higher resolution traits would have to be down-sampled to match the resolution of the lowest resolution traits. There is also an extension to deal with this which can be explored in the future (Kutz et al. 2015).

## Conclusion

Longitudinal predictions from genomic data are agronomically important as they allow for a shortening of the breeding cycle and resources for phenotyping, which represent significant bottlenecks in modern plant breeding (Crossa et al. 2017). Typically, in time-dependent genomic prediction studies, accuracy is assessed using the snapshot method quantified by the Pearson correlation between measured and predicted values separately at each time point. In the present study, we additionally examined longitudinal accuracy and MSE—the Pearson correlation and MSE between the true and predicted trait development trajectories over multiple time points. We compared the performance of a powerful multi-trait genomic prediction method, MegaLMM, in several configurations, with that of a recently introduced method, dynamicGP, in predicting a multi-trait time series of 50 traits. The performance of the two approaches was assessed using both snapshot and longitudinal accuracies. We found that the tested configurations of MegaLMM outperformed dynamicGP in terms of both snapshot and longitudinal accuracy across all time points. We further examined the characteristics of well predicted versus poorly predicted trait developmental trajectories, revealing weak associations. Our findings were observed in a single data set from a single species, and further research is required testing the generality of these observations on other data sets. Furthermore, our study points at the need for additional methodological developments to tackle the challenging problem of multi-trait temporal prediction from genetic markers and for integrating single measurement agronomically relevant traits, such as yield into longitudinal models.

## Supplementary Information

Below is the link to the electronic supplementary material.

Supplementary file 1 (pdf 6878 KB)

Supplementary file 2 (xlsx 77 KB)

## Data Availability

Genotypic and phenotypic data for maize are available at 10.5281/zenodo.14959484.

## Code availability

All codes that were used to generate the results of this study are freely available at https://github.com/dobby978/dynamicGP-MegaLMM.
