# Supplementary material for: Comparative analysis of genomic prediction approaches for multiple time-resolved traits in maize
Source: Theor Appl Genet. 2026 Feb 6;139(2):63. doi: 10.1007/s00122-026-05162-4 (PMC12881034; doi:10.1007/s00122-026-05162-4)
Supplement: Supplementary file 1 — (pdf 6878 KB) [file 122_2026_5162_MOESM1_ESM.pdf]

Supplementary Figures

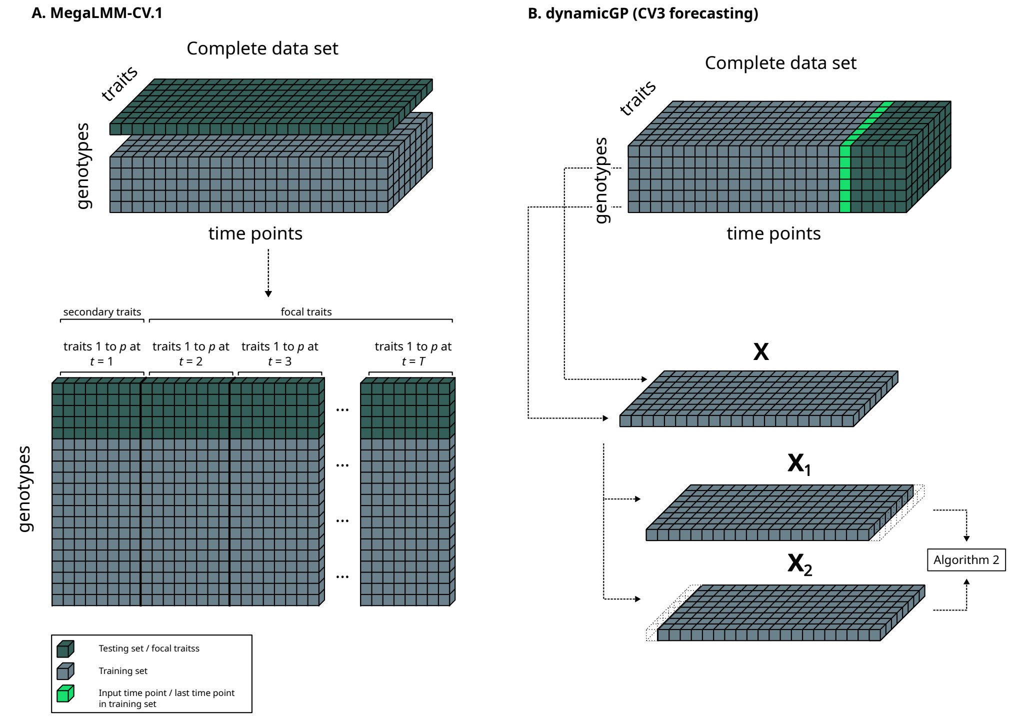

Fig. S1: **Illustration of the data usage in the compared MegaLMM formulations. A. MegaLMM-CV1** In the first comparison, we used 80% of the data to train MegaLMM and employed them to predict the remaining 20% of data. **B. CV3** dynamicGP models trained on the first 20 time points and used to predict the final 5 time points. The 20th time point was used as the initial input time point.

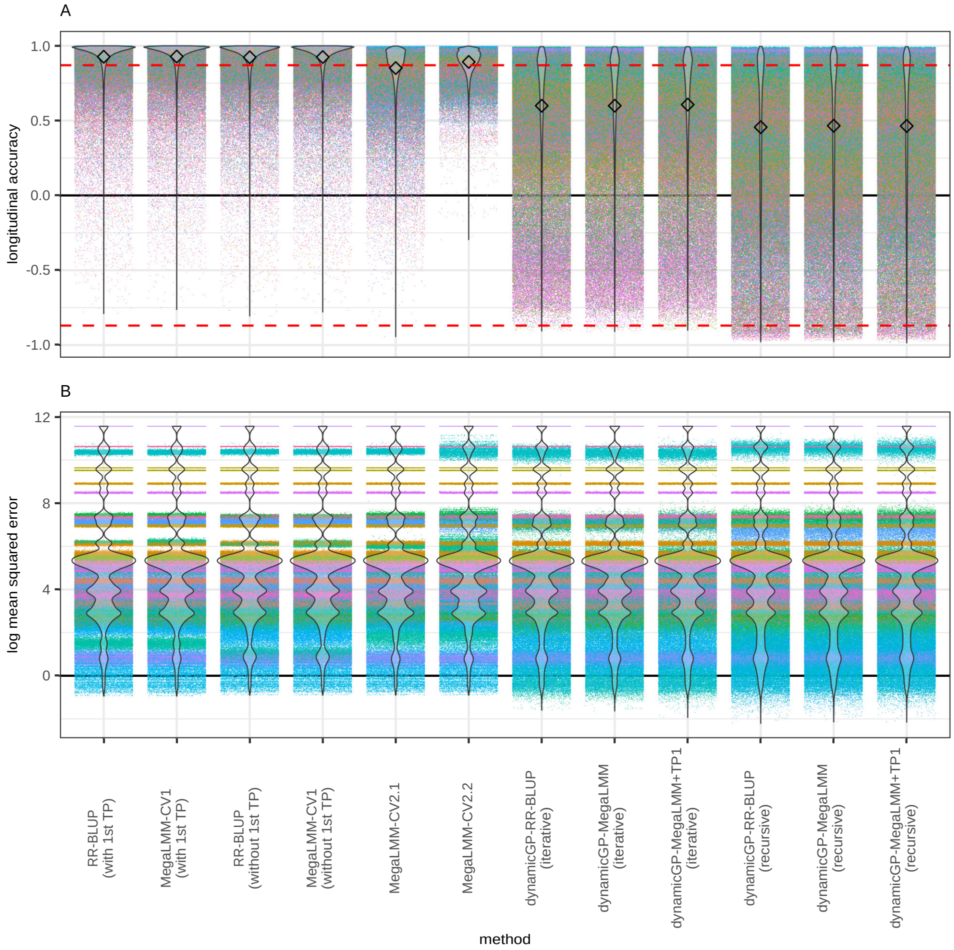

Fig. S2: **Classical GP models have highest longitudinal accuracy. A.** Accuracy of predicted trait dynamics along time series aggregated across all cv-iterations and -folds, traits, and genotypes for univariate RR-BLUP models for each trait-time-point pair and MegaLMM-CV1, with and without the first time point, MegaLMM-CV2.1 and MegaLMM-CV2.2 as well as both iterative and recursive variants of dynamicGP-RR-BLUP, dynamicGP-MegaLMM, dynamicGP-MegaLMM+TP1. Accuracy was assessed as the Pearson correlation between true and predicted values. All values above the red dashed lined are significant after Bonferroni multiple testing correction. Diamonds indicate mean prediction accuracy for each method.**B.** Log-transformed mean squared error of predicted trait dynamics along time series aggregated across all CV iterations and -folds, traits, and genotypes for the six methods. Within each method there are a total of 330,000 tests performed, corresponding to the total number of combinations of 50 traits over 330 genotypes and 20 iterations of cross-validation, with each indicated by a point. Colors in **A.** & **B.** indicate different traits.

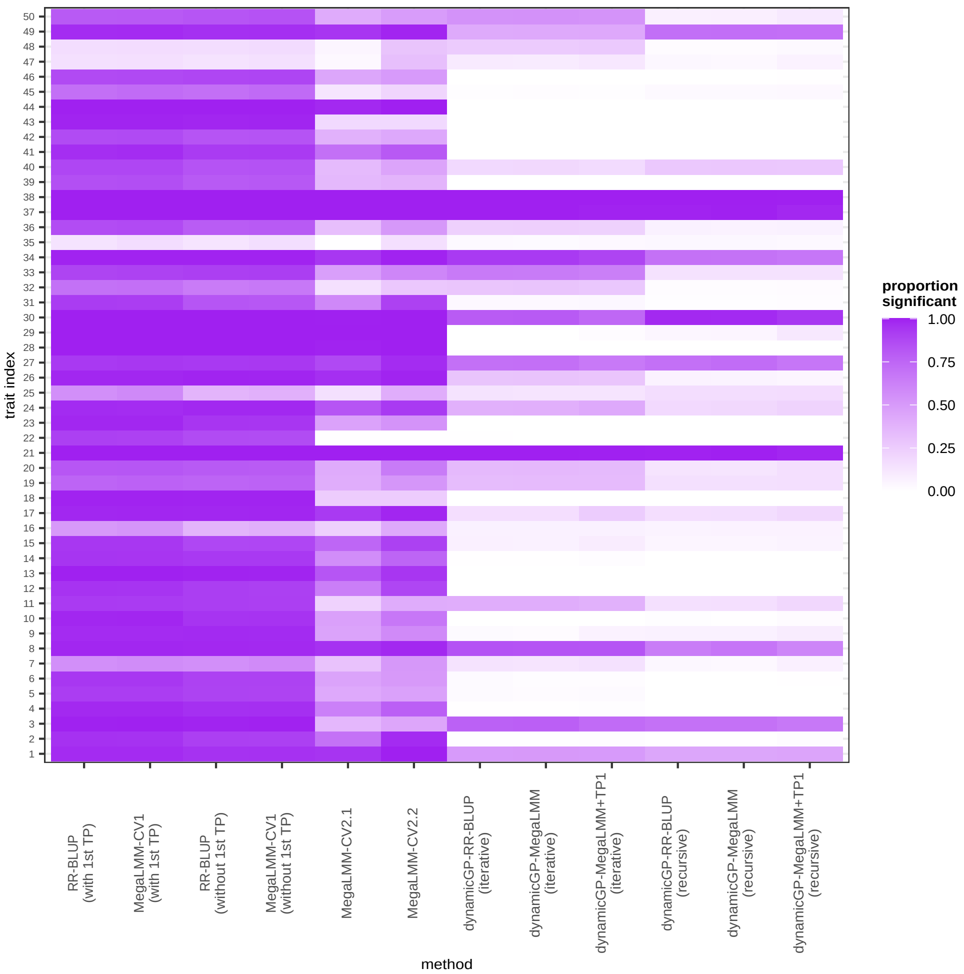

Fig. S3: **Classical GP models have highest proportion of significant positive predictive correlations.** Proportion of significant positive predictive correlations after Bonferroni multiple testing correction for each trait using each of the twelve methods. Table S1 contains mapping of traits to index.

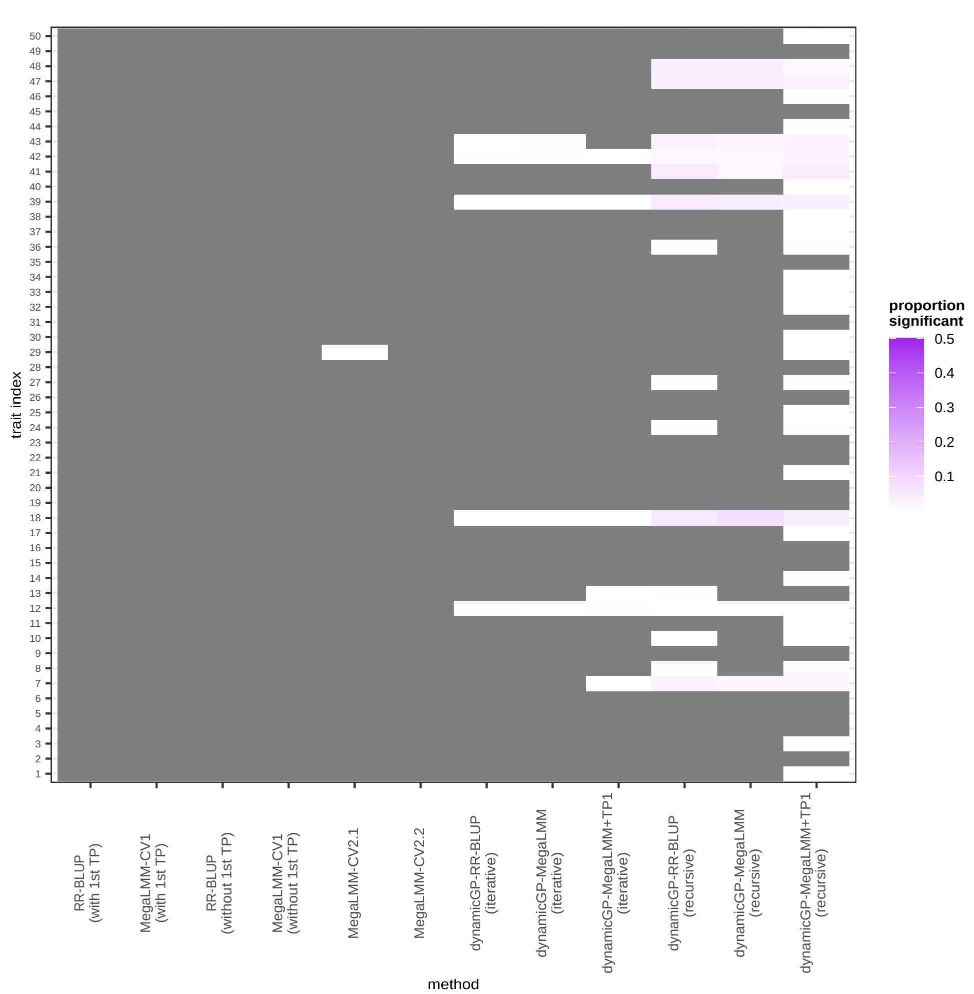

Fig. S4: **Univariate RR-BLUP models have highest proportion of significant negative predictive correlations.** Proportion of significant negative predictive correlations after Bonferroni multiple testing correction for each trait using each of the twelve methods. Grey filled cells indicate 0 significant correlations to highlight the relatively low proportions. Supplementary Table 1 contains mapping of traits to index.

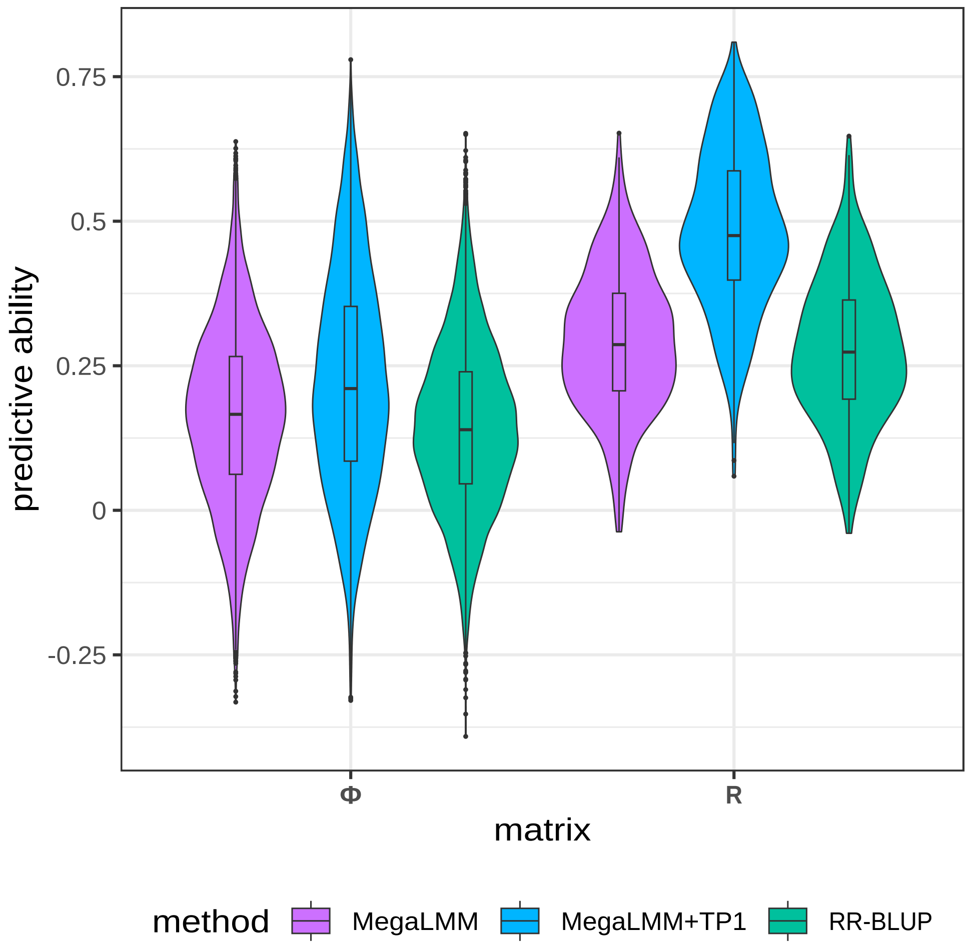

Fig. S5: **Usage of phenomic initial state increases predictive ability of matrix elements of $\mathbf{\Phi}$ and R. a.** Distributions of matrix elements of $\mathbf{\Phi}$ and $\mathbf{R}$ predicted using RR-BLUP, MegaLMM, and MegaLMM with the initial phenomic state as secondary traits (MegaLMM+TP1).

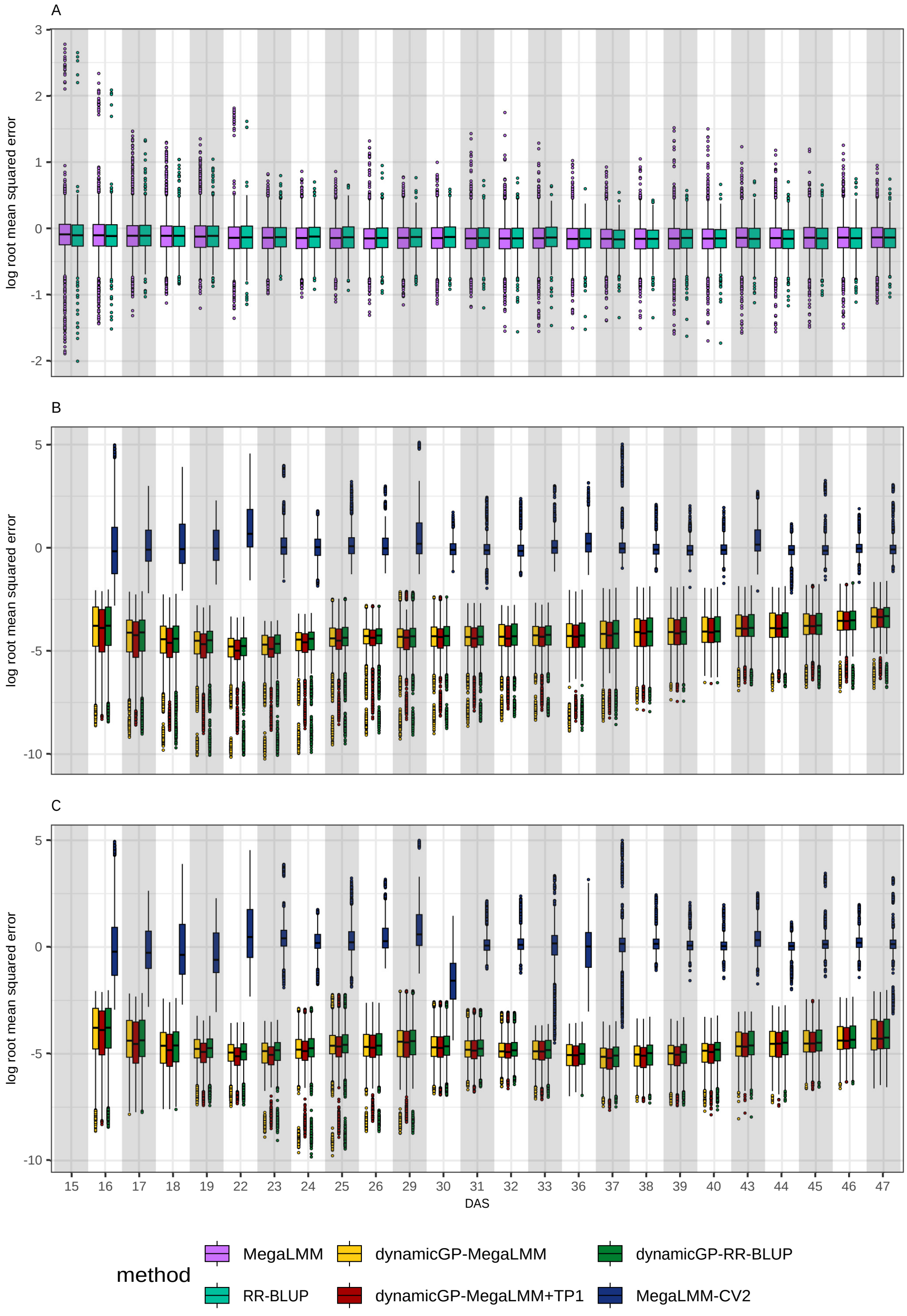

A
B
C
log root mean squared error
DAS
15
16
17
18
19
22
23
24
25
26
29
30
31
32
33
36
37
38
39
40
43
44
45
46
47
method
MegaLMM
RR-BLUP
dynamicGP-MegaLMM
dynamicGP-MegaLMM+TP1
dynamicGP-RR-BLUP
MegaLMM-CV2

Fig. S6: **Comparative analysis of MegaLMM and dynamicGP variants based on mean squared error (MSE). A.** Mean time-point-specific snapshot MSE across all traits for RR-BLUP models in ST-STP configuration and MegaLMM models in MT-MTP configuration with no secondary traits. **B. & C.** Mean time-point-specific snapshot MSE for dynamicGP models using either RR-BLUP or MegaLMM as core method for $\mathbf{\Phi}$ and $\mathbf{R}$ prediction. DynamicGP-MegaLMM was tested both with (+TP1) and without the initial phenotypic state included as secondary traits. **B.** Recursive configuration and MegaLMM-CV2 with the initial phenotypic state as secondary traits to predict the remainder of the time-series. **C.** Iterative configuration with MegaLMM-CV2 with the phenotypic state at a single time point as secondary traits to predict the following time point.

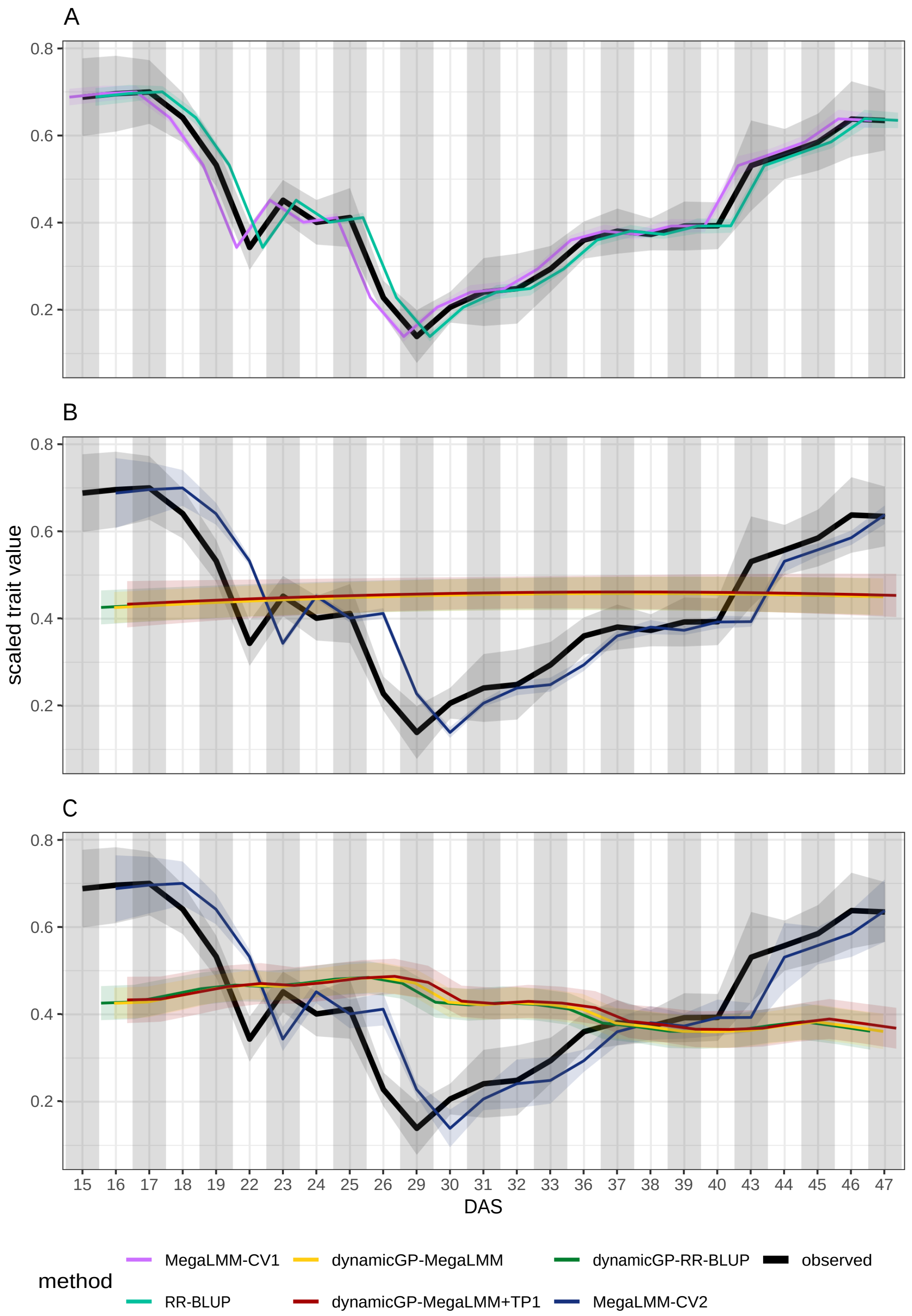
A
B
C
0.8
0.6
0.4
0.2
scaled trait value
15 16 17 18 19 22 23 24 25 26 29 30 31 32 33 36 37 38 39 40 43 44 45 46 47
DAS
method
MegaLMM-CV1
RR-BLUP
dynamicGP-MegaLMM
dynamicGP-MegaLMM+TP1
dynamicGP-RR-BLUP
MegaLMM-CV2
observed

Fig. S7: **Comparative analysis of MegaLMM and dynamicGP variants based on predicted developmental trajectories.** Predicted trajectory of the trait with the lowest mean longitudinal accuracy across all of the tested methods (*top.intensity.fluo.intensity.phenol.mean*) by (**A.**) ST-STP RR-BLUP models, and MegaLMM-CV1 models; three versions of the (**B.**) recursive configuration of dynamicGP (RR-BLUP, MegaLMM, MegaLMM+TP1), and MegaLMM-CV2.1; and (**C.**) corresponding iterative versions of dynamicGP and MegaLMM-CV2.2. Predicted trajectory is the mean predicted value of a given trait at each time point across all cross-validation iterations and folds, shading represents standard deviation.

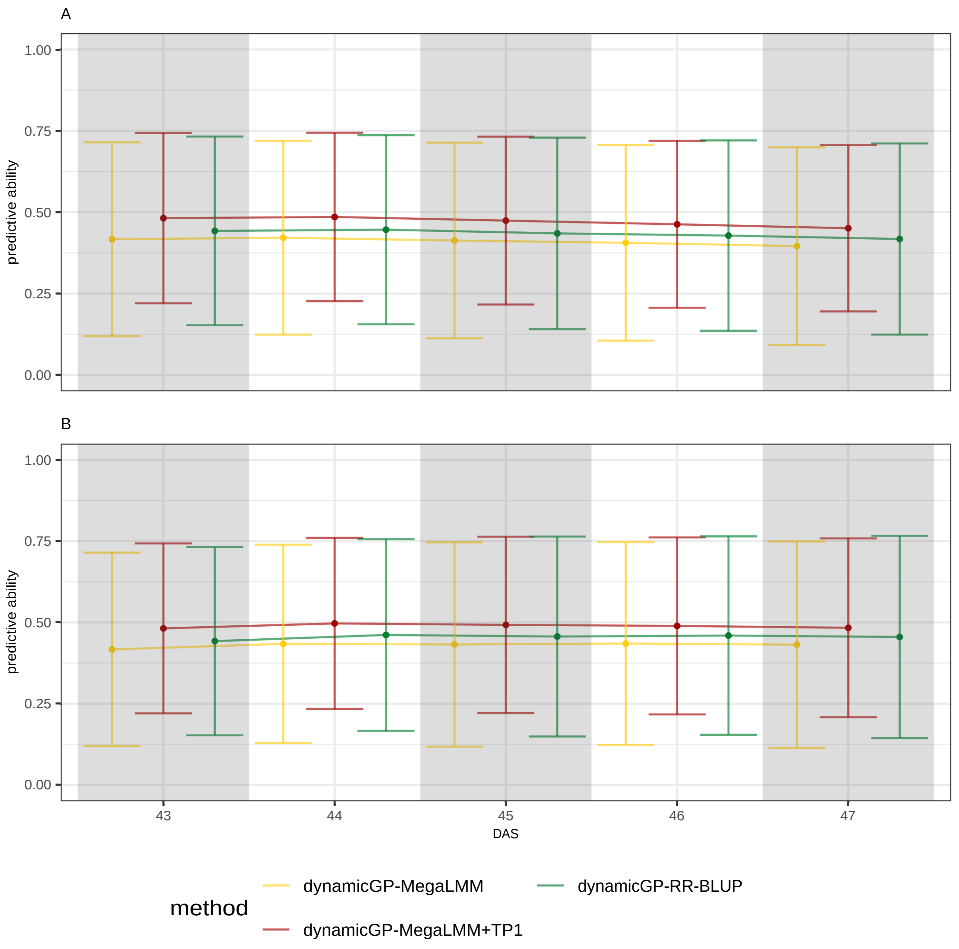

Fig. S8: **Comparative analysis of dynamicGP variants in forecasting the final five time points.** Mean time-point-specific snapshot PCC for dynamicGP models trained on the first 20 time points and predicting the final 5 time points using either RR-BLUP or MegaLMM as core method for $\mathbf{\Phi}$ and $\mathbf{R}$ prediction. DynamicGP-MegaLMM was tested both with (+TP1) and without the initial phenotypic state included as secondary traits. **A.** Recursive configuration and MegaLMM-CV2 with the initial phenotypic state as secondary traits to predict the remainder of the time-series. **B.** Iterative configuration with MegaLMM-CV2 with the phenotypic state at a single time point as secondary traits to predict the following time point.

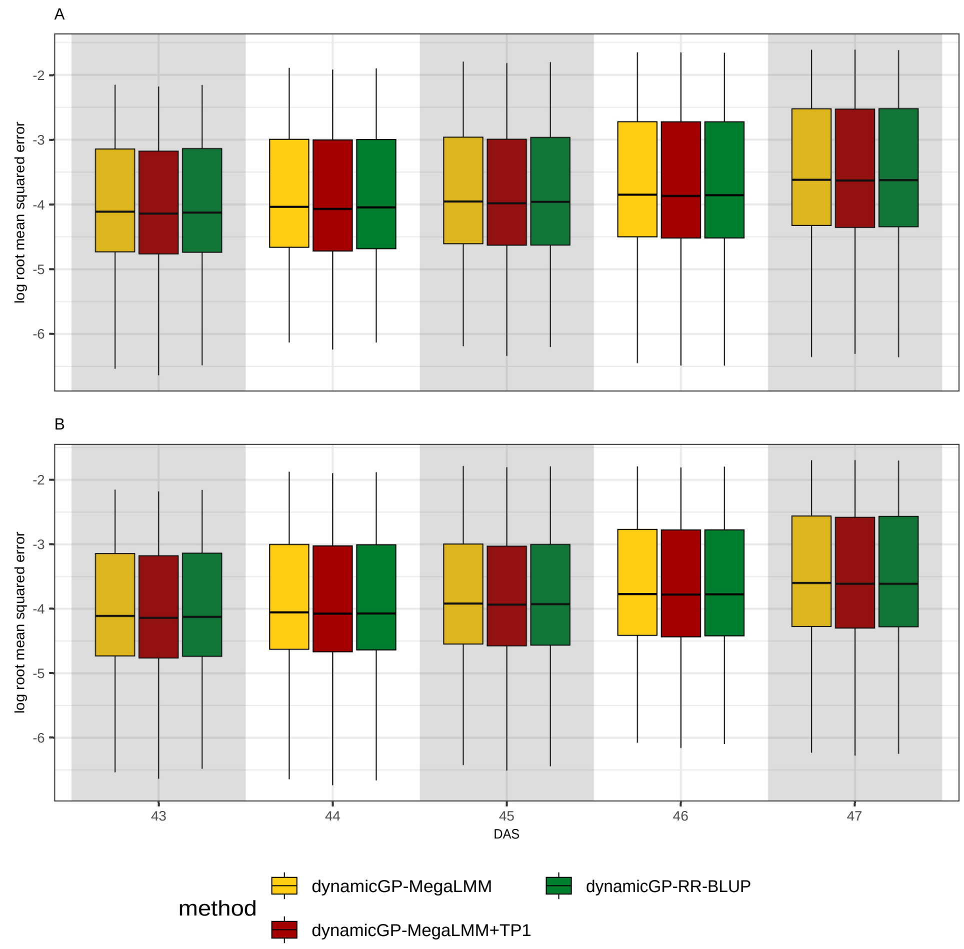

Fig. S9: **Comparative analysis of dynamicGP variants in forecasting the final five time points with mean squared error (MSE).** Mean time-point-specific snapshot MSE for dynamicGP models trained on the first 20 time points and predicting the final 5 time points using either RR-BLUP or MegaLMM as core method for $\mathbf{\Phi}$ and $\mathbf{R}$ prediction. DynamicGP-MegaLMM was tested both with (+TP1) and without the initial phenotypic state included as secondary traits. **A.** Recursive configuration and MegaLMM-CV2 with the initial phenotypic state as secondary traits to predict the remainder of the time-series. **B.** Iterative configuration with MegaLMM-CV2 with the phenotypic state at a single time point as secondary traits to predict the following time point.

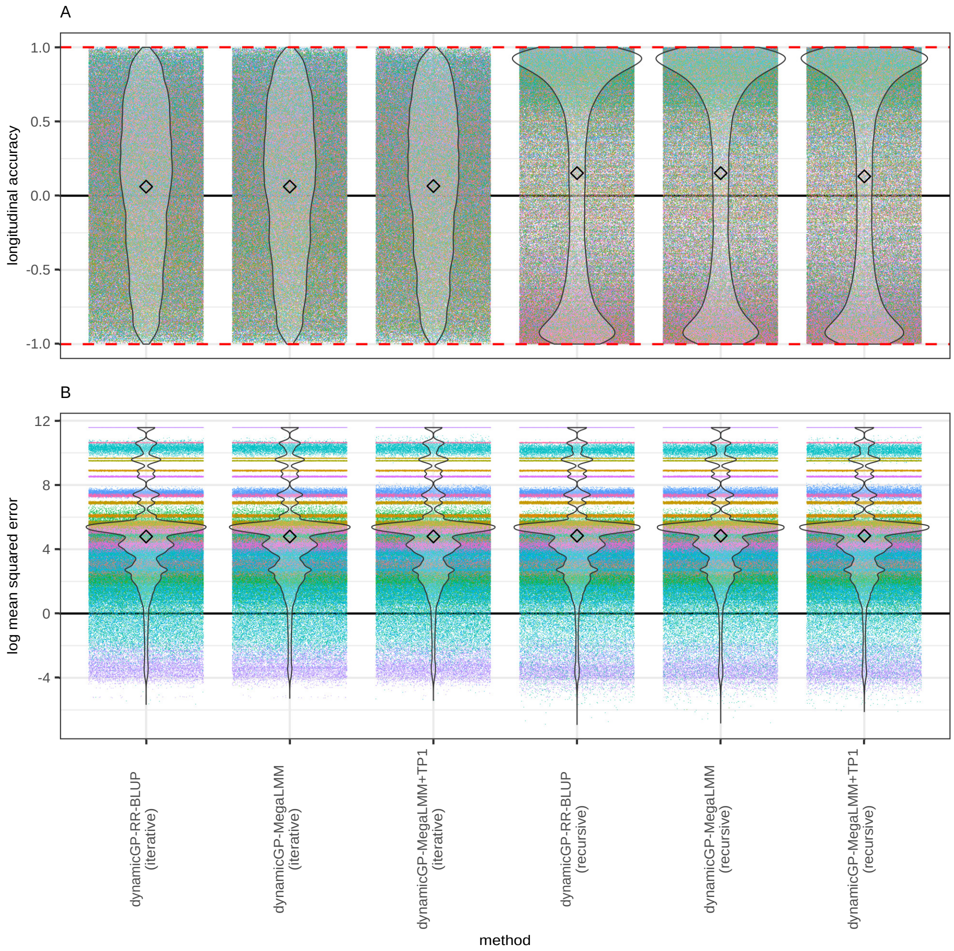

Fig. S10: **Classical GP models have highest longitudinal accuracy. A.** Accuracy of predicted trait dynamics for dynamicGP models trained on the first 20 time points and predicting the final 5 time points along time series aggregated across all cv-iterations and -folds, traits, and genotypes for univariate RR-BLUP models for each trait-time-point pair and MegaLMM-CV1, with and without the first time point, MegaLMM-CV2.1 and MegaLMM-CV2.2 as well as both iterative and recursive variants of dynamicGP-RR-BLUP, dynamicGP-MegaLMM, dynamicGP-MegaLMM+TP1. Accuracy was assessed as the Pearson correlation between true and predicted values. All values above the red dashed lined are significant after Bonferroni multiple testing correction. Diamonds indicate mean prediction accuracy for each method.**B.** Log-transformed mean squared error of predicted trait dynamics along time series aggregated across all CV iterations and -folds, traits, and genotypes for the six methods. Within each method there are a total of 330,000 tests performed, corresponding to the total number of combinations of 50 traits over 330 genotypes and 20 iterations of cross-validation, with each indicated by a point. Colors in **A.** & **B.** indicate different traits.
